# A Network of Paralogous Stress Response Transcription Factors in the Human Pathogen *Candida glabrata*

**DOI:** 10.3389/fmicb.2016.00645

**Published:** 2016-05-09

**Authors:** Jawad Merhej, Antonin Thiebaut, Corinne Blugeon, Juliette Pouch, Mohammed El Amine Ali Chaouche, Jean-Michel Camadro, Stéphane Le Crom, Gaëlle Lelandais, Frédéric Devaux

**Affiliations:** ^1^Laboratoire de Biologie Computationnelle et Quantitative, Centre National de la Recherche Scientifique, Institut de Biologie Paris-Seine, UMR 7238, Sorbonne Universités, Université Pierre et Marie CurieParis, France; ^2^École Normale Supérieure, Paris Sciences et Lettres Research University, Centre National de la Recherche Scientifique, Institut National de la Santé et de la Recherche Médicale, Institut de Biologie de l'École Normale Supérieure, Plateforme GénomiqueParis, France; ^3^Centre National de la Recherche Scientifique, UMR 7592, Institut Jacques Monod, Université Paris Diderot, Sorbonne Paris CitéParis, France; ^4^Évolution, Centre National de la Recherche Scientifique, Institut de Biologie Paris-Seine, UMR 7138, Sorbonne Universités, Université Pierre et Marie CurieParis, France

**Keywords:** yeast, Yap, ChIP-seq, transcriptome, regulatory networks, evolution

## Abstract

The yeast *Candida glabrata* has become the second cause of systemic candidemia in humans. However, relatively few genome-wide studies have been conducted in this organism and our knowledge of its transcriptional regulatory network is quite limited. In the present work, we combined genome-wide chromatin immunoprecipitation (ChIP-seq), transcriptome analyses, and DNA binding motif predictions to describe the regulatory interactions of the seven Yap (Yeast AP1) transcription factors of *C. glabrata*. We described a transcriptional network containing 255 regulatory interactions and 309 potential target genes. We predicted with high confidence the preferred DNA binding sites for 5 of the 7 CgYaps and showed a strong conservation of the Yap DNA binding properties between *S. cerevisiae* and *C. glabrata*. We provided reliable functional annotation for 3 of the 7 Yaps and identified for Yap1 and Yap5 a core regulon which is conserved in *S. cerevisiae, C. glabrata*, and *C. albicans*. We uncovered new roles for CgYap7 in the regulation of iron-sulfur cluster biogenesis, for CgYap1 in the regulation of heme biosynthesis and for CgYap5 in the repression of *GRX4* in response to iron starvation. These transcription factors define an interconnected transcriptional network at the cross-roads between redox homeostasis, oxygen consumption, and iron metabolism.

## Introduction

*Candida glabrata* is a unicellular yeast from the Hemiascomycetes phylogenetic group and a commensal host of the human mucosal microbiota. However, in patients with severe immunodeficiency, it can cause invasive systemic infections, with high mortality rates (about 40–60%). While *Candida albicans* remains the main cause of systemic candidiasis (50–70%), *C. glabrata* ranks second (20–25%), and its prevalence has increased in the last decades (Perlroth et al., 2007). An important prerequisite to the acquisition of virulence traits in *C. glabrata* is its ability to adapt and be resistant to environmental variations, which allows the pathogen to colonize many different niches and organs in the human body, with very different features in terms of pH, temperature, redox potential, iron, zinc, or oxygen availability, etc. (Domergue et al., 2005; Nevitt and Thiele, 2011). Moreover, *C. glabrata* is able to survive and grow in the harsh environment of the phagolysosomes of macrophages (Seider et al., 2011, 2014).

The mechanisms that allow these adaptations rely partially on transcriptional regulatory networks. Systematic combination of transcriptome analyses of loss of function mutants for different transcription factors and of genome-wide chromatin immunoprecipitation has led to the comprehensive description of genome-wide transcriptional regulatory networks in the model yeast *Saccharomyces cerevisiae* (Babu et al., 2004; Harbison et al., 2004; Teixeira et al., 2014). These global approaches have also been extensively used in *C. albicans*. In this species, more than 75 specific transcription factors (over a total of about 150) have been analyzed by genome-wide approaches and comprehensive network-based models are available for ribosome biogenesis, iron homeostasis, or biofilm formation (Lavoie et al., 2010; Chen et al., 2011; Nobile et al., 2012; Fox et al., 2015). In contrast, the global transcriptional regulation in *C. glabrata* has been poorly studied. As of 2016, only 10 transcription factors have been analyzed on a genome-wide scale in this species (Vermitsky et al., 2006; Lelandais et al., 2008; Roetzer et al., 2008, 2011; Kuo et al., 2010a; Caudle et al., 2011; Ferrari et al., 2011; Miyazaki et al., 2013; Noble et al., 2013; Paul et al., 2014; Merhej et al., 2015; Wu et al., 2015). While *C. glabrata* and *C. albicans* share a common ecological niche and are from the same genus, they are very distant species according to their genomic sequence, and their ability to successfully infect humans involves quite different strategies (Brunke and Hube, 2013). For instance, in contrast to *C.albicans*, the ancestor of *C.glabrata* and *S.cerevisiae* experienced a whole-genome duplication event. In addition, *C.albicans* is usually diploid, switching frequently from yeast to hyphal growth under stress conditions, while *C.glabrata* is strictly haploid and grows mostly in the yeast form. Therefore, the *C. glabrata* transcriptional networks cannot be simply inferred from the knowledge acquired in *C. albicans* (Gabaldon and Carrete, 2015).

In the present work, we conducted a network-based analysis of the seven transcription factors belonging to the Yap (Yeast AP1) family in *C. glabrata*. The Yap proteins belong to the pap subfamily of bZIP transcription factors and are homologous to the CREB, ATF2, and Fos/Jun transcription factors of vertebrates (Fujii et al., 2000; Reinke et al., 2013). The model yeast *S. cerevisiae* has 8 Yap members (named ScYap thereafter; Supplementary File S1), most of which are involved in adaptation to environmental changes (Rodrigues-Pousada et al., 2010). ScYap1 is the major regulator of oxidative stress responses caused by reactive oxygen species (ROS), metals and drugs (reviewed in Rodrigues-Pousada et al., 2010). ScYap2 (also named Cad1), the ohnolog of Yap1, is involved in cadmium resistance (Hirata et al., 1994; Azevedo et al., 2007; Mazzola et al., 2015). The role of ScYap3 is unknown but it has been shown to contribute to the resistance to benzenic compounds and to 6-Nonadecynoic acid (North et al., 2011; Xu et al., 2012; Adeboye et al., 2014). The ScYap4 (also named Cin5) and ScYap6 ohnologs are involved in salt stress response (Mendizabal et al., 1998; Nevitt et al., 2004; Ni et al., 2009). ScYap5 is responsible for the activation of the high iron stress response (Li et al., 2008; Pimentel et al., 2012). ScYap7, the ohnolog of ScYap5, was recently found to be a repressor of the nitric oxide oxidase encoding gene *YHB1* (Merhej et al., 2015) and ScYap8 is involved in the response to arsenic (Bobrowicz et al., 1997; Amaral et al., 2013; Kumar et al., 2015).

*C. glabrata* has 7 Yap members, which slightly differ from the *S. cerevisiae* family (Supplementary File S1). Orthologues of Yap1 (*CAGL0H04631g*), Yap2 (*CAGL0F03069g*, named CgYap1 thereafter), Yap5 (*CAGL0K08756g*, named CgYap5 thereafter), and Yap7 (*CAGL0F01265g*, named CgYap7 thereafter) are present, but Yap8 is absent. Two versions of Yap3 (*CAGL0K02585g* and *CAGL0M10087g*, named CgYap3a and CgYap3b thereafter) and only one ortholog for the Yap4 and Yap6 pair (*CAGL0M08800g*, named CgYap4/6 thereafter) are found. Only three of these Yaps have been studied previously in *C. glabrata*. CgYap1 has been shown to be involved in oxidative stress response, with a set of targets which is significantly conserved compared to ScYap1, but with different DNA binding preferences (Chen et al., 2007; Cuellar-Cruz et al., 2008; Lelandais et al., 2008; Kuo et al., 2010a; Goudot et al., 2011; Roetzer et al., 2011). CgYap7 has been shown to have a conserved role in nitric oxide oxidase repression (Merhej et al., 2015). Finally, as in *S. cerevisiae*, CgYap5 is involved in the activation of the *CCC1* and *GRX4* genes under high iron conditions (Merhej et al., 2015). In the present work, we conducted chromatin immunoprecipitation experiments followed by high-throughput sequencing (ChIP-seq) and transcriptome analyses to determine the targets for the seven Yap transcription factors of *C. glabrata*. The CgYap network included 309 genes and 255 regulatory interactions. From these results, we could predict with high confidence the preferred DNA binding sites for 5 of the 7 CgYaps and show a strong conservation of the Yap DNA binding properties between *S. cerevisiae* and *C. glabrata*. We provided functional annotation for 3 of the 7 CgYaps and identified for Yap1 and Yap5 a core regulon which is conserved in *S. cerevisiae, C. glabrata*, and *C. albicans*. Our data pointed out new roles for CgYap7 in the regulation of iron-sulfur cluster biogenesis, for CgYap1 in the regulation of heme biosynthesis and for CgYap5 in the repression of *GRX4* in response to iron starvation.

## Materials and methods

### Strains

The list of the strains used in this study is available in Supplementary File S2. All the strains were derived from the ΔHTU parental strain (Kitada et al., 1995). The genomic myc-tagging and deletion of the different *CgYAP* was performed as described previously (Merhej et al., 2014, 2015). Briefly, deletion or myc-tagging cassettes were PCR amplified from the M. Longtine's plasmids (Longtine et al., 1998) with oligonucleotides containing in 5′ homology sequences flanking the desired genomic insertion points. At least 10 micrograms of purified PCR product was used to transform ΔHTU cells using a standard yeast transformation protocol (Merhej et al., 2015). Genotyping of the clones growing on selective media was done by PCR. The PCR-verified clones for the knock-out were then verified by southern blot (Merhej et al., 2015). The correct myc-tagging of the *CgYAP* was verified by sequencing of the gene and western blot (Merhej et al., 2015). All the oligonucleotides used for cassette preparation and genotyping are listed in Supplementary File S2.

### Yeast cultures and growth conditions

All cultures were conducted in a rotative shaker at 30°C in YPD (Glucose 2%, yeast extract 1%, Bactopeptone 1%). Stress conditions used were: 1 mM sodium selenite, 1 M NaCl, 2 mM cadmium chloride, 5 mM iron sulfate, or 0.5 mM bathophenanthroline disulfonate (BPS). These doses were chosen, based on preliminary microarray and growth assay experiments, to induce a transcriptional response in the wild type without causing significant differences of growth rates between the wild type and the *CgYAP* knock-out mutants (data not shown).

### Chromatin immunoprecipitation and high-throughput sequencing

For ChIP, myc-tagged *CgYAP* strains were grown in YPD until exponential phase (OD = 0.8) and then stressing agents were added for 30 min. Cross-linking of the cells and ChIP were performed as described previously (Lelandais et al., 2016). The parental ΔHTU (untagged strain) was grown and processed the same way to provide the mock-IP samples. Sequencing of the IPs, Input DNAs and mock IPs samples and primary data analyses (quality controls and mapping of the reads) were performed as described previously (Lelandais et al., 2016). All experiments were performed twice and the reads of the replicate averaged before the peak calling step, except for CgYap5 for which one of the two replicates had poor read coverage and was not used for further analyses. Peak calling was performed with the bpeaks software (Merhej et al., 2014), using both the Input DNA and the mock IP as references. For peak calling using the Input DNA as reference, the bpeaks parameters were T1 = 2, T2 = 2, T3 = 1.5, T4 = 0.7. For peak calling using the Mock IP as reference, the bpeaks parameters were T1 = 2, T2 = 2, T3 = 1.5, and T4 = 0. Only the peaks which were found by the two analyses were kept for further processing. These peaks were then manually checked on a genome browser (Thorvaldsdottir et al., 2013) to discard artifactual peaks (e.g., peaks centered on a tRNA locus or perfectly overlapping a highly expressed ORF) which would have escaped the bpeaks filter (Supplementary Files S3, S4). The ChIP seq data can be downloaded from the GEO database (accession number: GSE77904).

### Transcriptome analyses

Knock-out and wild type strains were grown in 50 mL of YPD until exponential phase (OD = 0.8) and then stressing agents were added. After 30 min, 20 mL of each cell cultures were flash-frozen in two volumes of cold ethanol and collected by centrifugation. The OD of the cultures were monitored before the stress treatment and every 30 min for 2 h after stress treatment. Only samples from wild type and knock-out cultures which showed similar growth rates (±10%) (Thompson et al., 2013) were used for transcriptome comparisons. Total RNA was extracted, quality controlled and quantified as described previously (Merhej et al., 2015). One microgram of total RNA was used for fluorescent cDNA synthesis according to the amino-allyl protocol (Merhej et al., 2015). The cDNA were labeled with Cy3 and Cy5 and hybridization was performed as previously described (Merhej et al., 2015). Two biologically independent experiments were performed for each condition, using dye switch. We used custom *C. glabrata* Agilent arrays in an 8 × 60 k format (array express accession number: A-MEXP-2402). After overnight hybridization and washing, the slides were scanned using a 2-micron Agilent microarray scanner. The images were analyzed using the feature extraction software (Agilent technologies) and normalized using global LOESS (Lemoine et al., 2006). The mean of the biological replicates was calculated. A gene was considered as being differentially expressed if its mean absolute Log2(fold change) value was more than 0.75 and if its expression variation was considered as being statistically significant using the LIMMA package with a cut-off *p*-value of 0.02 (Ritchie et al., 2015). The complete microarray data are available at Array express database under the accession number: E-MTAB-4457.

### TFBS predictions

DNA sequences of ChIP peaks were retrieved from their genomic locations (BED file) using the “getfasta” function from the BEDTOOLS suite (Quinlan and Hall, 2010). These genomic sequences were used as inputs for the “peak-motif” tool to search for regulatory motifs (Thomas-Chollier et al., 2012). An additional filtering step was added to the standard peak motif procedure to discard low complexity motifs (e.g., CCCCCCC) or motifs which were found in < 20% of the peaks (Supplementary File S5).

### Network building

The ChIP peaks were assigned to genes as described previously (Merhej et al., 2014). When a peak was located in a divergent promoter (i.e., an intergenic region in between two divergent genes) the two genes were fused in one target in the network named “gene 1/gene 2,” unless we had transcriptome evidence supporting the regulation of one of the two genes. In this case, only the name of the regulated gene was kept. The network was represented using the igraph library (igraph.org, R programming language; Csardi and Nepusz, 2006), combining three different types of information (Supplementary Table 1). The ChIP parameter was used to define interactions (arrows) between the different Yap factors and their target promoters. The transcriptome parameter was used to color arrows depending on the directionality of the regulation (activation, repression, or no detected change). The TFBS parameter was used to color target promoters depending of the presence of the identified TFBS in the corresponding ChIP peaks.

### Gene Ontology and gene set enrichment analyses

GO analyses were performed using the “GO term finder” tool at the CGD database, with default parameters (Inglis et al., 2012). GSEA were performed using the GSEA module of the Jexpress software suite with a cut-off FDR of 1% (Subramanian et al., 2005; Stavrum et al., 2008). We used as input files transcriptome analyses of the responses of *C. glabrata* wild type cells to fluconazole (Kuo et al., 2010b), sodium salt (Roetzer et al., 2008; Wapinski et al., 2010), heat shock (Roetzer et al., 2008; Wapinski et al., 2010), hydrogen peroxide (Wapinski et al., 2010; Roetzer et al., 2011), menadione (Roetzer et al., 2011), glucose starvation (Roetzer et al., 2008), sorbic acid (Jandric et al., 2013), iron excess and iron starvation (this work, E-MTAB-4457), cadmium chloride and sodium selenite (Thiebaut et al., unpublished data).

## Results

### The Yap network in *C. glabrata*

To identify the gene targets and characterize the regulatory interactions of the seven Yap transcription factors of *C. glabrata*, we used three different approaches. First, we performed ChIP-seq experiments using myc-tagged versions of each factor. For CgYap1, CgYap2, CgYap4/6, and CgYap5, we submitted the corresponding tagged strains to stress conditions known to induce full activity of their *S. cerevisiae* orthologues, i.e., oxidative stress caused by a metalloid (namely selenium) for Yap1 (Haugen et al., 2004; Salin et al., 2008), cadmium for Yap2 (Azevedo et al., 2007; Mazzola et al., 2015), salt excess for Yap4/6 (Nevitt et al., 2004; Ni et al., 2009), and iron excess for Yap5 (Li et al., 2008; Pimentel et al., 2012). For CgYap7, which was shown to have a constitutive activity (Merhej et al., 2015), the experiments were performed in standard growth conditions. For the two orthologs of Yap3, whose role remains unknown in *S. cerevisiae*, the experiments were performed using cells grown in YPD and cells exposed to a pleiotropic stress inducer (selenite; Salin et al., 2008). The ChIP-seq data were analyzed taking as a reference both the input control and the mock IP control, to sort out a maximum of the false positive peaks due to highly expressed loci (Park et al., 2013; Teytelman et al., 2013). Second, we used the ChIP-seq data to predict the preferred Transcription Factor DNA binding sites (TFBS) for each CgYap. Reciprocally, we identified all the ChIP-peaks which contain the predicted consensus in their promoter sequence. Third, we compared the transcriptome of wild type and null mutants for each CgYap, using the same growth conditions as those used for ChIP-seq experiments. Hence, we identified the genes for which expression was altered, directly or indirectly, in the absence of the corresponding transcription factor.

**Figure 1:**
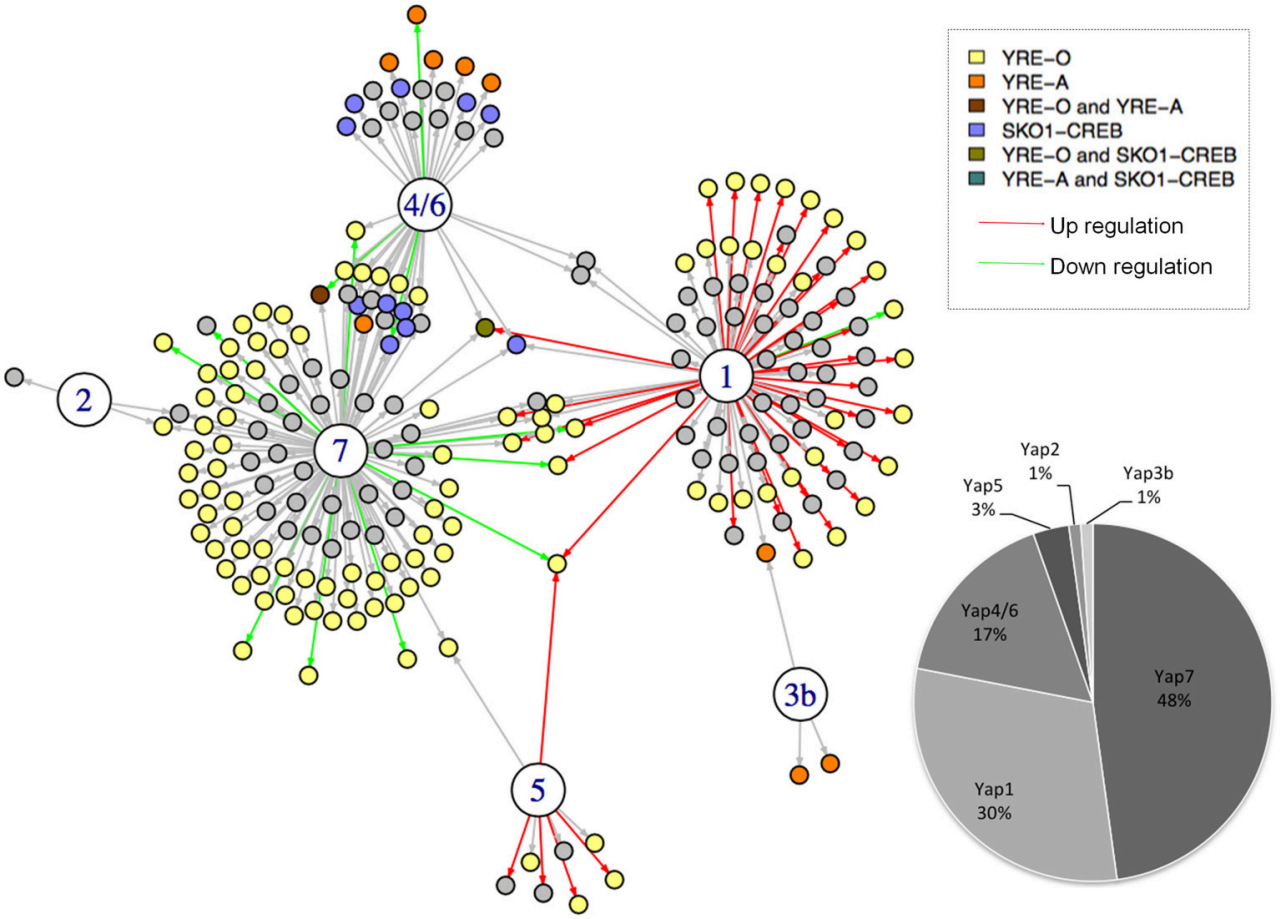
**The Yap network of ***Candida glabrata*****. The large circles represent the CgYap transcription factors (1 = CgYap1; 2 = CgYap2, etc.) and the small circles are the potential targets. An arrow indicates a potential regulatory interaction based on ChIP-seq. The color of the arrow indicates the directionality of the potential regulation based on transcriptome data (red, positive regulation; green, negative regulation; black, no change detected). The color of the targets indicates the consensus DNA sequences detected in the ChIP-peaks (see color code on the upper right). The percentage of targets for each CgYap is indicated on the bottom right. This network was represented using I-GRAPH and the information from Supplementary Table 1.

To build a network from these three sources of information, we used a scoring system based on simple but meaningful logical rules (Supplementary Table 1). Briefly, a regulatory interaction was included in the network if it was detected by ChIP-seq. Then, the interactions and the edges were differently labeled depending on the transcriptome and TFBS data (Figure 1). As a consequence, the different interactions in the final network do not have the same value, depending on whether they were supported by one, two, or three experimental evidences (Supplementary Table 1). The final CgYap network contained 6 transcription factors and 255 regulatory interactions involving 214 promoters and 309 potential target genes (Figure 1). We could not identify any target gene for CgYap3a, neither by ChIP-seq or by transcriptome analyses. Notably, 62% of the interactions in the network were supported by at least two evidences (Figure 1). The majority of the ChIP targets that we identified had only one peak in their promoter. However, in few cases, several binding sites could be unambiguously detected for CgYap1 and CgYap7 (Supplementary File S6). The number of interactions for each CgYap was highly variable, from 3 for CgYap2, up to 118 for CgYap7 (Figure 1). Relatively few redundancies were observed between the different Yaps, only 38 genes (18% of the edges) are targeted by more than one CgYap.

### *C. glabrata* Yap transcription factor binding sites

**Figure 2:**
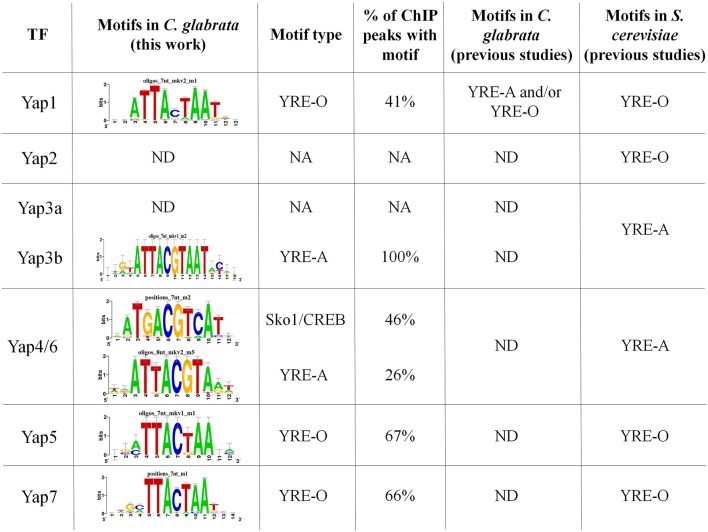
**CgYaps Transcription Factor Binding Sites predictions based on ChIP peaks**. The motifs were predicted from the ChIP-peaks sequences using Peak Motif (Thomas-Chollier et al., 2012). The motifs previously found for the Yap transcription factors in *S. cerevisiae* and for CgYap1 in *C. glabrata* are indicated (Tan et al., 2008; Kuo et al., 2010a; Goudot et al., 2011). The complete Peak Motif predictions are available in Supplementary File S5.

As mentioned above, the peaks identified from ChIP-seq data were used to predict the TFBS for 5 of the 7 studied CgYap (Figure 2). In *S. cerevisiae*, the Yap proteins were classified in two categories based on their preferred TFBS (named YRE for Yap Response Elements): ScYap1, ScYap2, ScYap5, and ScYap7 bind to YRE-O (TTACTAA) motifs while ScYap3, ScYap4, and ScYap6 rather recognize YRE-A (TTACGTAA) motifs (Tan et al., 2008; Kuo et al., 2010a). We found that this dichotomy was remarkably conserved in *C. glabrata* (Figure 2). The predicted binding sites for CgYap1, CgYap5 and CgYap7 were very close to the perfect YRE-O consensus. In contrast, the YRE-A motif was enriched in the ChIP peaks of CgYap3b and CgYap4/6. These YRE motifs were identified as the best predicted motifs for all Yaps, except for CgYap4/6 (Figure 2, Supplementary File S5). For this transcription factor, the best identified motif was ATGACGTCAT, which differs from the canonical YRE-A motif by its higher GC content and which actually corresponds to the consensus motif published for another bZIP subfamily, the CREB/ATF2 factors (Fujii et al., 2000). Interestingly enough, in *S. cerevisiae* this motif was associated to the Sko1 transcription factor, which is a yeast homolog of the ATF2/CREB factors (Pascual-Ahuir et al., 2001; Gordan et al., 2011). Sko1 has been shown to contribute to the salt stress response of *S. cerevisiae*, together with ScYap4 and ScYap6, and to share a large number of targets with these factors (Ni et al., 2009).

The proportion of ChIP peaks containing the best predicted TFBS was remarkably high (from 41% for Yap1 up to 100% for Yap3b) (Figures 1, 2). This provided a posteriori confirmation that our ChIP-seq analyses procedure efficiently filtered out the false positive peaks.

### Directionality of the CgYap activities

Because we included transcriptome data in our network, we could determine the directionality (i.e., activation or repression) of some of the regulatory interactions set up by the different Yap proteins (Figure 1). This allowed us to predict the activator or repressor nature of these CgYaps. We observed that, in the conditions that were studied (selenite, excess of iron, salt stress, or YPD, respectively), CgYap1 and CgYap5 were strict activators, while CgYap4/6 and CgYap7 were strict repressors (Figure 1). The deletion of CgYap2 and CgYap3b had no effect on their targets in the conditions that we studied and therefore we had no information on their activity. The overlaps between transcriptome results and ChIP-seq results were remarkably high in the sense that, taking into account the directionality mentioned above, between 40 and 90% of the expression changes observed in the transcriptome analyses involved genes to which promoters were bound by the TF according to ChIP-seq data (Supplementary File S7). The reciprocal was not true for CgYap7 and CgYap4/6, for which most of the ChIP targets showed unchanged expression in the mutant. This may be due either to functional redundancies for the regulation of these genes or because our transcriptome experiments were not conducted in conditions in which these regulations were active.

### Functional annotation of the Yap network

**Figure 3:**
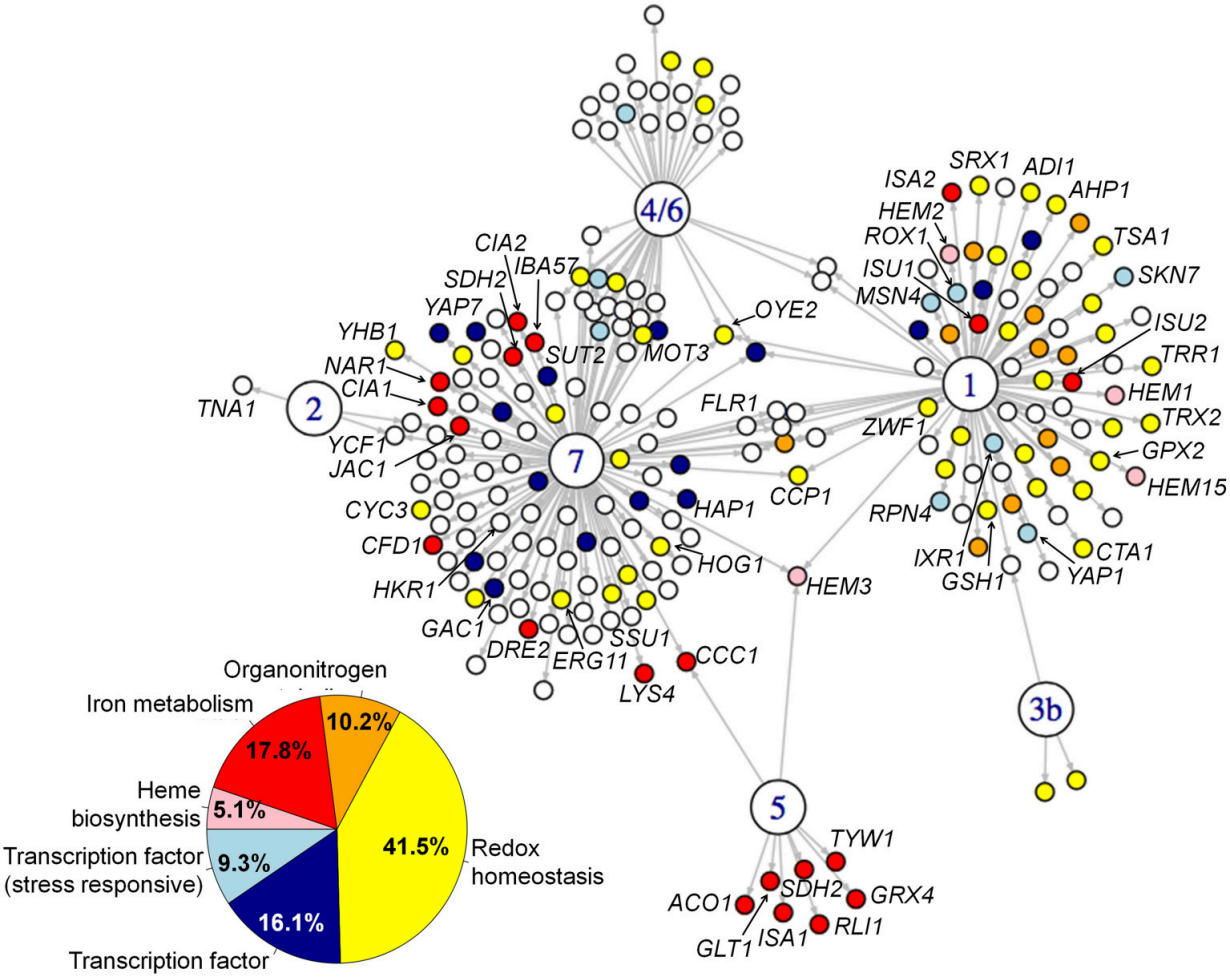
**Functional annotation of the CgYap network**. Gene ontology analyses were performed on the target sets of the CgYap network. The main enriched categories are represented here by the colors of the corresponding targets (color code on the bottom left). “Oxido-reduction” corresponds to the GO categories “oxidation-reduction process,” “response to oxidative stress,” and “oxidoreductase activity.” “Transcription factor” corresponds to the GO category “sequence-specific DNA binding.” “transcription factor (stress responsive)” corresponds to the GO category “regulation of transcription from RNA polymerase II promoter in response to stress.” “Heme biosynthesis” corresponds to the GO category “heme biosynthetic process.” “iron metabolism” corresponds to the GO categories “iron-sulfur cluster assembly,” “iron-sulfur cluster binding” and “iron ion homeostasis.” “Organitrogen” corresponds to the GO category “organonitrogen compound metabolic process.” The complete GO results are available in Supplementary Table 1. The names of the genes which are discussed in the main text are indicated. The phenotypes of the *CgYAP* mutant strains in various stress conditions are shown in Supplementary File S10.

We performed Gene Ontology (GO) term enrichment analyses on the whole set of genes in the network and on the individual sets of target of each CgYap (Figure 3, Supplementary Table 1). The whole network was enriched in GO categories related to oxido-reduction processes and iron homeostasis (Figure 3). CgYap1, CgYap5, and CgYap7 were the main contributors to these categories, while CgYap2, CgYap3b, and CgYap4/6 did not show any significant enrichment. More specifically, CgYap1 was the main contributor of targets related to oxidative stress response, oxido-reduction and chemical stress response (e.g., *TRR1, TRX2, OYE2, GPX2, TSA1, CTA1, SRX1*, …). Its target set was also enriched in genes involved in heme biosynthesis (*HEM1, HEM3, HEM15*, and *HEM2*). CgYap5 target list was clearly associated to iron sulfur cluster binding (*ISA1, TYW1, ACO1, RLI1, SDH2, GLT1*) and iron homeostasis (*GRX4, CCC1*). The only GO category to be enriched in CgYap7 targets was iron sulfur cluster metabolism. This includes genes involved in the cytosolic and mitochondrial iron sulfur assembly pathway (*CIA1, CIA2, DRE2, NAR1, CFD1, IBA57, JAC1*) and in iron sulfur cluster binding (*LYS4, SDH2*). CgYap7 also targets 10 genes encoding oxidoreductases (*CCP1, ERG11, YHB1, OYE2*, etc…) and genes encoding heme containing proteins (*YHB1, CCP1*) or related to heme metabolism (*HEM3, CYC3*). The targets of CgYap1 and CgYap7 included many genes encoding transcription factors. For instance, several transcription factors involved in oxygen homeostasis and oxidative stress responses are targeted by CgYap1 (*ROX1, MSN4, RPN4, SKN7, IXR1*). Remarkably, CgYap1 and CgYap7 both bound their own promoter, suggesting auto-regulation (Supplementary Table 1).

### Conservation of the CgYap1/5/7 subnetwork

**Figure 4:**
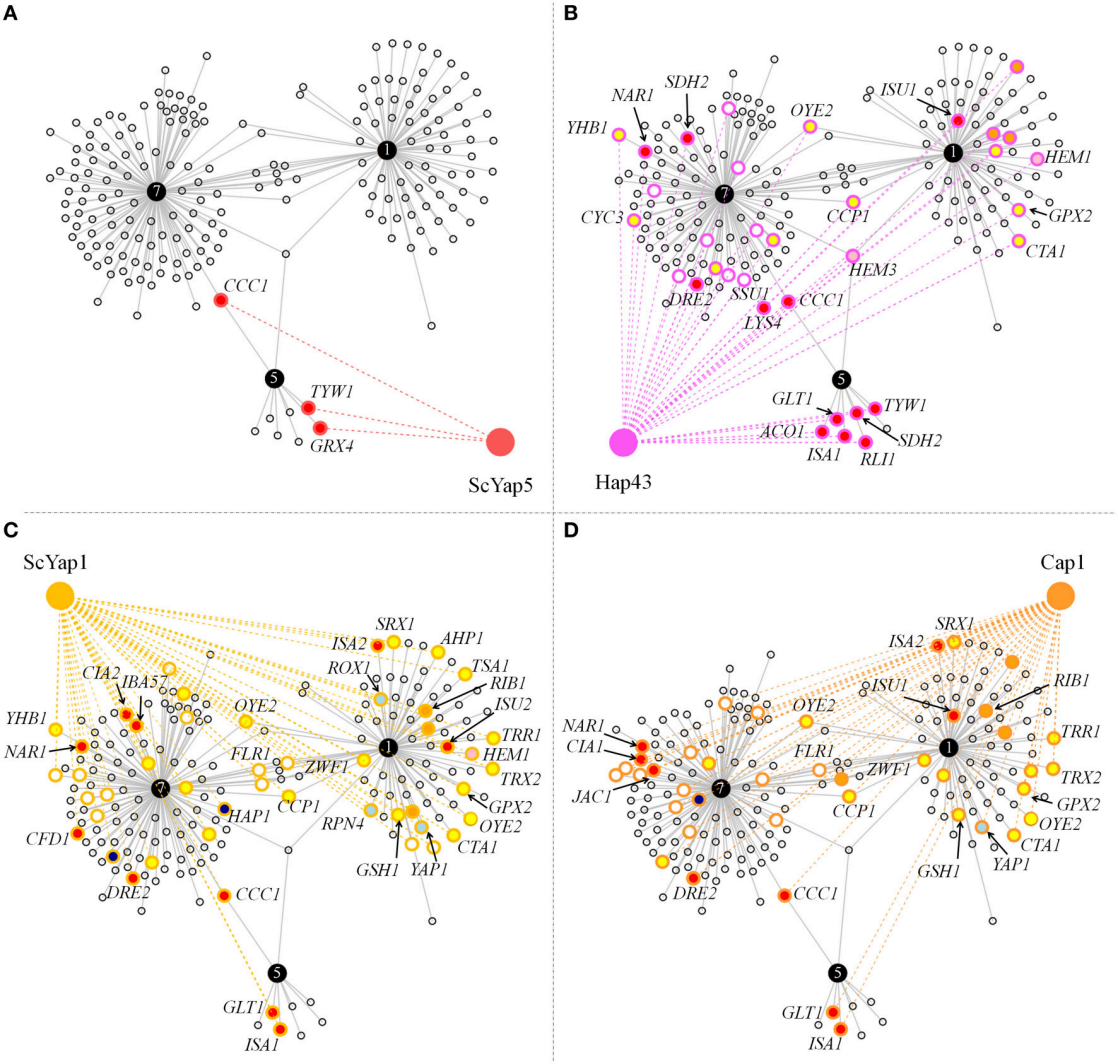
**Conservation of the CgYap1/5/7 subnetwork in ***S. cerevisiae*** and ***C. albicans*****. Graphs represent the targets of CgYap1, CgYap5, and CgYap7 which were also shown to be regulated by ScYap5 **(A)**, Hap43 **(B)**, ScYap1 **(C)**, and Cap1 **(D)**. The color code for the gene targets is the same as for Figure 3. Only the GO code of the conserved targets is shown. The names of the genes which are discussed in the main text are indicated. The data used to draw this figure are available in Supplementary Table 2.

The functional annotation presented above pointed out a CgYap1/CgYap5/CgYap7 network centered on iron and redox homeostasis. To assess the conservation of this sub-network we compared the targets of CgYap1, CgYap5, and CgYap7 with the targets of their orthologues in *S. cerevisiae* and *C. albicans* (Li et al., 2008, 2011; Salin et al., 2008; Znaidi et al., 2009; Chen et al., 2011; Hsu et al., 2011; Singh et al., 2011; Pimentel et al., 2012; Supplementary Table 2). The functional categories in which Yap1 and Yap5 are involved were remarkably conserved: ScYap1 and Cap1 are also involved in oxido-reduction processes (Salin et al., 2008; Znaidi et al., 2009), ScYap5 and Hap43 are involved in iron homeostasis, but ScYap5 is an activator of iron stress response while Hap43 has a role in repressing iron consuming genes in iron limiting conditions (Li et al., 2008; Hsu et al., 2011; Singh et al., 2011). No clear GO category could be attributed to ScYap7 besides its role in *YHB1* repression but all previous genome-wide studies (for instance Harbison et al., 2004) have been conducted in a strain background in which the *ScYAP7* gene is interrupted by a frame-shift mutation (Merhej et al., 2015). In terms of gene targets, the three Yap5 targets which had been validated in *S. cerevisiae* (*CCC1, GRX4*, and *TYW1*; Li et al., 2008, 2011; Pimentel et al., 2012) were conserved in *C. glabrata* (Figure 4A). Remarkably, despite the large evolutionary distance between *C. glabrata* and *C. albicans*, all CgYap5 targets, except *GRX4*, are targets of its orthologue Hap43 (Figure 4B). Hap43 also shares 19 targets with CgYap7, many of which are involved in iron sulfur cluster metabolism, redox homeostasis or heme metabolism (e.g., *NAR1, DRE2, LYS4, SDH2, SSU1, OYE2, YHB1, CCP1, HEM3*, …).

Among the 82 targets that we identified for CgYap1, 28 are orthologous to targets of ScYap1 and 20 to Cap1 targets (Figures 4C,D). Of these, 15 were common to the three orthologs. This set of highly conserved Yap1 targets includes several enzymes known to play important roles in redox balance (*OYE2, TRX2, TRR1, CTA1, GPX2, GSH1, ZWF1, CCP1*), the MFS permease *FLR1* (named *MDR1* in *C. albicans*) and, remarkably, Yap1 itself, suggesting that the auto-regulation of Yap1 may actually play a significant role in its function. Besides this relatively high conservation of the Yap1 regulon in yeasts, a remarkable specificity of CgYap1 is its role in the direct regulation of several genes encoding enzymes of the heme biosynthetic pathway, a feature which was documented neither in *S. cerevisiae* nor in *C. albicans*.

### Overlap between the CgYap network and stress responses in *C. glabrata*

**Figure 5:**
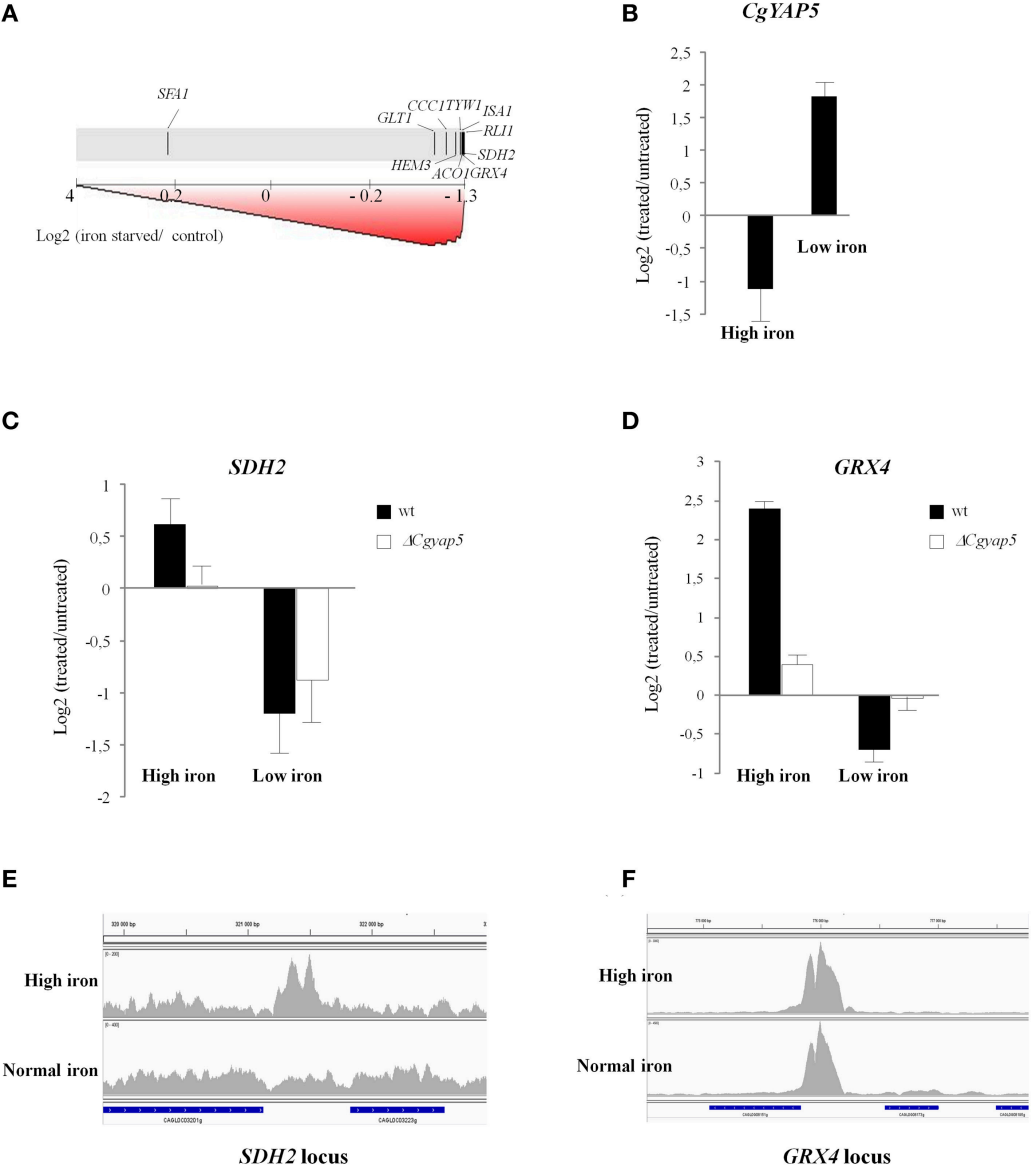
**CgYap5 acts as a repressor of ***GRX4*** in iron starvation. (A)** Graphical output of the Gene Set Enrichment Analyses, using as a gene set the targets of CgYap5 identified in our study and using as a test dataset the response of wild type *C. glabrata* cells to iron starvation caused by 0.5 mM BPS. The transcriptome data are symbolized by the gray scale with the most induced genes on the left and the most repressed on the right. The positions of the CgYap5 targets are indicated on this scale by black vertical lines. **(B)** The expression of *CgYAP5* is inversely correlated to iron concentration. Histograms based on microarray analyses of the *C. glabrata* response to high iron concentration and to iron starvation caused by BPS. **(C,D)** *SDH2* and *GRX4* in response to high iron or to iron starvation, in wild type (black bars) or Δ*Cgyap5* (white bars) cells. The impact of the *CgYAP5* deletion on the repression of *GRX4* in low iron conditions was confirmed by Q-RT-PCR (Supplementary File S11). **(E,F)** Binding of CgYap5 to the promoters of *SDH2* and *GRX4* in high iron or in normal iron (YPD media) conditions (ChIP-seq).

We next used Gene Set Enrichment Analyses (GSEA; Subramanian et al., 2005) to look for enrichments of our CgYap target sets in transcriptome analyses of *C. glabrata* wild type cells to various environmental stresses. The list and origin of the transcriptome data which were used can be found in the methods. As could have been expected from the results presented in the previous chapters, the CgYap1 targets were significantly enriched among the genes induced by oxidative stress causing agents (H2O2, selenite, iron, with the exception of menadione; Supplementary File S8), supporting the general role of this transcription factor in the oxidative stress response of *C. glabrata* (Lelandais et al., 2008; Roetzer et al., 2011). More surprisingly, the targets of CgYap1 were enriched in heat shock and sorbic acid stress responses. Interestingly enough, the HSE (Heat Shock Element) was found to be present in 25% of the CgYap1 ChIP peaks (Supplementary File S5). In *S. cerevisiae*, previous works have identified connections between Yap1 activity on one hand and Hsf1 and some gene regulatory modules induced by heat shock on the other (Hahn et al., 2006; Wu and Li, 2008; Nussbaum et al., 2014). Our results suggest that these connections might be conserved in *C. glabrata*. In contrast, the targets of CgYap4/6 were not particularly enriched in osmotic stress responses caused by NaCl excess (Supplementary File S8), which questions the conservation in *C. glabrata* of the role of the ScYap4 and ScYap6 in this process. As expected, the targets of CgYap5 were globally induced by iron excess in *C. glabrata* but we also observed that the CgYap5 set of targets was significantly repressed in iron depleted conditions caused by BPS treatment or selenite exposure (Supplementary File S8, Figure 5A). Moreover, our transcriptome analyses of iron starvation and iron excess responses in wild type *C. glabrata* cells showed that *CgYAP5* itself had expression levels which were inversely correlated to the iron concentration (Figure 5B), suggesting an active role for this transcription factor in iron starvation. Obviously, the fact that CgYap5 targets were repressed by iron starvation did not necessarily mean that CgYap5 was directly involved in this regulation. For instance, in *S. cerevisiae*, the repression of many iron consuming genes such as *ACO1, SDH2, ISA1*, and *CCC1* in iron starved conditions occurs pos-transcriptionally and is mediated by the RNA binding proteins Cth1 and Cth2 (Puig et al., 2005). To assess the actual role of CgYap5 in the iron starvation response of *C. glabrata*, we analyzed the transcriptome response of Δ*Cgyap5* cells to BPS treatment and compared this response with the one of wild type cells. We observed that most of the genes, which were dependent on Yap5 for their high iron induction, were similarly repressed by BPS in the wild type and in the Δ*Cgyap5* mutant (exemplified by *SDH2* on Figure 5C), indicating that this repression was not CgYap5 dependent. The only exception was *GRX4*, for which repression was totally abolished when CgYap5 was absent (Figure 5D). Consistent with these results, ChIP-seq experiments conducted in iron-replete conditions showed that the binding of CgYap5 to *GRX4* promoter was constitutive, while binding to its other targets was detected only at high iron concentrations (Figures 5E,F). Notably, the *GRX4* expression level was previously shown to be independent of CgYap5 in standard growth conditions (Merhej et al., 2015), indicating that the effect detected here was indeed specific of iron starvation. These results strongly suggest that, in addition to its role in the iron stress response, CgYap5 plays an active role in the iron starvation response by directly repressing the expression of *GRX4*.

## Discussion

### A methodology to build highly consistent regulatory networks

Global ChIP and transcriptome analyses are powerful tools to achieve comprehensive descriptions of large transcriptional regulatory networks (Babu et al., 2004; Harbison et al., 2004). However, the interpretation of these networks is dampened by the tendency of these techniques to generate large numbers of false positives. For instance, highly expressed genomic regions (tRNA genes, glycolytic enzymes encoding genes, etc.) have been shown to be nonspecifically enriched in ChIP-seq experiments, leading to tens to hundreds of misidentified “targets” (Park et al., 2013; Teytelman et al., 2013). This bias is better captured, but only partially corrected, using mock IP as reference for peak calling, rather than input DNA (Park et al., 2013; Krebs et al., 2014). For transcriptome analyses, relatively minor differences between wild type and mutant growth rates or stress response dynamics can eventually produce tens of differentially expressed genes which have no real relationship with the mutation being tested (Thompson et al., 2013). We took into account these previous observations and designed experimental and bioinformatics procedures in which, 1- peak calling was performed using both the input DNA and the mock IP as references to efficiently sort out peaks corresponding to tRNA or highly expressed ORFs loci; and 2- only wild type and mutant cell cultures having very similar growth rates before and after the stress treatment were compared in transcriptome analyses. These simple precautions led to a final network showing unusually high consistency between ChIP results and TFBS predictions on the one hand, and ChIP and transcriptome results on the other hand. Indeed, between 40 and 100% of the ChIP peaks identified contained one or several YRE (Yap Response Element). For comparison, the rate of YRE containing peaks in previous ChIP-chip studies conducted on the Yaps of *S. cerevisiae* (Tan et al., 2008; Ni et al., 2009) or on Yap1 in *C. glabrata* (Kuo et al., 2010a) ranged from 15 to 30%. Similarly, between 40 and 90% of the genes for which the transcriptome data showed an expression change had a ChIP peak in their promoter. This percentage ranged from 0 to 25% in a previous study of the *S. cerevisiae* Yap family (Tan et al., 2008).

These data allowed us to propose targets with a reasonably high level of confidence for 6 of the 7 Yaps in *C. glabrata*, to predict the preferred binding motifs of 5 of them and identify enriched functional categories for CgYap1, CgYap5, and CgYap7.

### Conserved DNA binding properties of the Yap transcription factors

TFBS predictions based on the ChIP peaks pointed out a perfect conservation in the DNA binding properties of Yap1, Yap3b, Yap4/6, Yap5, and Yap7. As in *S. cerevisiae* (Tan et al., 2008), CgYap1, CgYap5, and CgYap7 were predicted to recognize preferentially the YRE-O site (TTACTAA) while CgYap3b and CgYap4/6 rather binds YRE-A (TTACGTAA). This result is consistent with the high conservation of the DNA binding domains of these proteins between *C. glabrata* and *S. cerevisiae* (Kuo et al., 2010a). Yet, this was unexpected in the case of CgYap1. Indeed, it was previously proposed, based on bioinformatic predictions from ChIP-chip data, that CgYap1 shifted its binding preferences from YRE-O to YRE-A due to a single mutation in the DNA binding domain compared to ScYap1 (Kuo et al., 2010a). This model was toned down by further analyses of the same dataset, which showed that YRE-O were as frequent as YRE-A in the promoters of CgYap1 targets (Goudot et al., 2011). Our ChIP-seq data unambiguously suggest that YRE-O is the preferred DNA binding site of CgYap1 and that the co-evolution scenario previously published has to be reconsidered. The discrepancy between these different studies may rely on the size of the genomic sequences which were used for TFBS predictions. Indeed previous ChIP-chip data provided peaks which were as wide as intergenic regions (800 base pairs in Goudot et al., 2011) while the binding regions identified by our ChIP-seq analyses for CgYap1 were 300 base pairs in average, leading to a much more precise identification of the actual binding location of the transcription factor (Supplementary File S4).

### The Yap1 core regulon

Hence, the DNA binding properties of Yap1 were remarkably conserved between *S. cerevisiae, C. glabrata*, and *C. albicans*, since the YRE-O was also shown to be the preferred binding motif of Cap1 (Znaidi et al., 2009; Goudot et al., 2011). What was true for DNA binding was also true at the level of gene targets and functional annotations. Indeed, our data confirmed the role of CgYap1 in redox homeostasis, as previously demonstrated (Chen et al., 2007; Lelandais et al., 2008; Kuo et al., 2010a; Roetzer et al., 2011). We identified a core Yap1 regulon of 28 targets between *S. cerevisiae* and *C. glabrata* and 15 conserved targets between *S. cerevisiae, C. glabrata*, and *C. albicans*. Notably, this enlarged by more than two-fold the list of conserved Yap1 targets which were previously identified (Kuo et al., 2010a; Goudot et al., 2011). This core Yap1 regulon is composed mostly of well-known and highly conserved actors of oxidative stress response such as gluthation peroxidase and gluthation synthetase, catalase, mitochondrial peroxydase, enzymes of the thioredoxin pathway (thioredoxins, thioredoxin reductase, thioredoxin peroxidase, and thioredoxin peroxydase reductase), the FLR1/MDR1 permease, enzymes involved in NADPH metabolism (NADPH oxydoreductase of the Old Yellow Enzyme family and glucose phosphate dehydrogenase). This confirms that, as suggested by previous work, oxidative stress response in general and the Yap1 control of this response in particular, do not fundamentally differ in *C. glabrata* compared with *S. cerevisiae* and *C. albicans* (Cuellar-Cruz et al., 2008; Lelandais et al., 2008; Kuo et al., 2010a; Gulshan et al., 2011; Roetzer et al., 2011; Briones-Martin-Del-Campo et al., 2014). Also, Yap1 binding to its own promoter, which had been demonstrated in *C. albicans* and *S. cerevisiae* (Salin et al., 2008; Znaidi et al., 2009), was conserved in *C. glabrata*. Although the primary activation of Yap1 has been shown to be at the post-translational level (Kuge et al., 1997; Zhang et al., 2000), this high conservation suggests that Yap1 autoregulation could play a role in conditions of acute oxidative stress, such as the ones used in the aforementioned studies. In support to this hypothesis, *ScYAP1, CgYAP1* and *CAP1* were all shown to be induced by oxidative stress at the mRNA level (Salin et al., 2008; Znaidi et al., 2009).

### Insights in the roles of Yap2 and Yap4/6 in *C. glabrata*

Only three ChIP targets could be detected for CgYap2 and CgYap3b, which did not allow us to propose functional annotations for these two factors. Still, it is interesting to notice that CgYap2 targets *YCF1*, which encodes a vacuolar transporter playing a key role in cadmium detoxification in *S. cerevisiae* (Wemmie et al., 1994; Li et al., 1997) and *TNA1*, the inactivation of which leads to cadmium sensitivity (Ruotolo et al., 2008). This suggests that the role of ScYap2 in cadmium resistance is conserved in *C. glabrata*. In *S. cerevisiae*, the activity of Yap2 is hidden by its partial functional redundancy with Yap1 (Azevedo et al., 2007; Iwai et al., 2010; Mazzola et al., 2015). Hence, it would be interesting to conduct Δ*Cgyap2* transcriptome analyses in a context in which *CgYAP1* has been knocked out.

We identified about 40 potential targets for CgYap4/6. The enrichment of the Sko1 binding motifs in the ChIP peaks indicated that the role of ScYap4 and ScYap6 in osmotic stress response may be conserved in *C. glabrata*. However, our GSEA and GO analyses showed that the CgYap4/6 targets were enriched neither in NaCl responsive genes nor in any particular functional category and the actual role of this factor remains to be elucidated. Our transcriptome analyses suggested that CgYap4/6 acts as a transcriptional repressor in the conditions that we tested. In *S. cerevisiae*, this point is controversial. ScYap4 and ScYap6 have been shown to recruit the general transcriptional repressor Tup1 (Hanlon et al., 2011) and bioinformatic analyses based on transcriptome data have suggested that they could be both repressors and activators (Tan et al., 2008). However, previous studies based on northern blots had shown that ScYap4 positively impacts the expression of three genes in response to osmotic shock (Nevitt et al., 2004).

### CgYap5 can act both as an activator and a repressor of glutaredoxin expression, depending on iron availability

Similarly to CgYap1, CgYap5 has a conserved role in the iron stress response. In *S. cerevisiae*, Yap5 acts at three levels against iron excess by 1- the induction of the glutaredoxin Grx4 which senses iron-sulfur clusters in the cytoplasm (Muhlenhoff et al., 2010) and negatively controls the activity of the Aft1/2 transcriptional activators of iron uptake (Ojeda et al., 2006; Ueta et al., 2012); 2- the induction of Ccc1 which transports cytoplasmic iron into the vacuole (Li et al., 2008); and 3- the overexpression of Tyw1 which is an iron-sulfur cluster containing protein and therefore contribute to iron sequestration (Li et al., 2011). We showed here that, in iron excess conditions, CgYap5 similarly controls the expression of *GRX4, CCC1*, and genes encoding proteins involved in iron sequestration either through iron-sulfur cluster binding and biogenesis (*TYW1, GLT1, ACO1, RLI1, SDH2, ISA1*) or through heme biosynthesis (*HEM3*).

**Figure 6:**
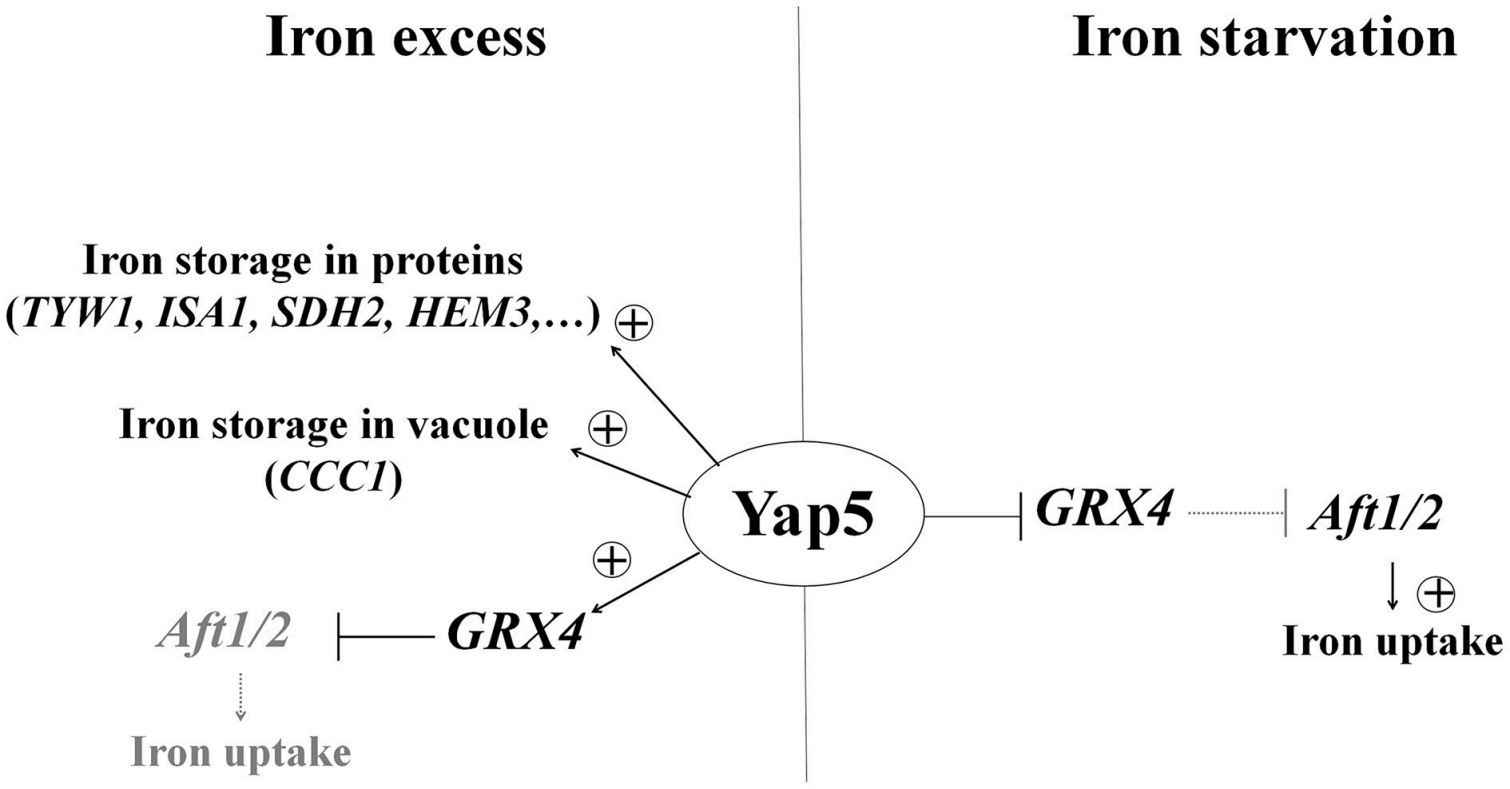
**A dual role for CgYap5 in iron excess and iron starvation**. In iron excess **(Left Panel)**, CgYap5 induces an iron stress response which is very similar to what was described in *S. cerevisiae*. In iron starvation **(Right Panel)**, CgYap5 represses *GRX4* expression and may indirectly contribute to the induction of iron uptake genes by the Aft1/2 transcription factors.

In addition to this conserved role in the detoxification of iron excess, we showed that CgYap5 is overexpressed in response to iron starvation and that it represses the expression of *GRX4* in these conditions. As mentioned above, Grx4 inhibits the iron starvation response by promoting the nuclear export of Aft1/2 when iron-sulfur clusters are abundant (reviewed in Lill et al., 2014). The regulation of glutaredoxin activity is mostly post-translational (Lill et al., 2014), but transcriptional repression of *GRX4* by CgYap5 may provide a supplementary layer of regulation to ensure full Aft1/2 activity in iron starvation conditions (Figure 6). This new role of Yap5 may be conserved in *S. cerevisiae*, since ScYap5 has been shown to bind the *GRX4* promoter independently of iron concentration and since the deletion of *ScYAP5* negatively impacts the nuclear localization of Aft1/2 in iron limiting conditions (Pimentel et al., 2012). However, the inactivation of *ScYAP5* does not seem to impact *GRX4* expression in iron-replete cells (Pimentel et al., 2012).

This dual role of CgYap5 is reminiscent of its HapX orthologs. HapX was initially identified as a key regulator of iron starvation response in filamentous ascomycetes (Hortschansky et al., 2007; Schrettl et al., 2010; Lopez-Berges et al., 2012), basidyomycetes (Jung et al., 2010), and hemiascomycetes of the *C. albicans* clade (where it is called Hap43; Hsu et al., 2011; Singh et al., 2011). HapX acts by repressing iron consuming genes when iron is limiting and HapX proteins are more expressed in iron starvation than in iron excess growth conditions (Singh et al., 2011; Gsaller et al., 2014). However, HapX was shown recently to play an additional role in activating the expression of some of its targets in response to iron excess in *Aspergillus* and *Fusarium sp*. (Gsaller et al., 2014). Interestingly, Yap5 and HapX both sense iron by directly binding iron-sulfur clusters through a conserved cysteine rich domain (CRD; Gsaller et al., 2014; Rietzschel et al., 2015). Our data indicate that Yap5 and HapX may have more in common than just this CRD. As indicated above, CgYap5 is also overexpressed and able to repress transcription in iron starved cells (Figure 6). Moreover, all the CgYap5 targets that we identified, except *GRX4*, are targets of Hap43. This conservation of targets is remarkable considering that Hap43 and Yap5 have different DNA binding properties: Hap43, like HapX in other fungi, mainly binds CCAAT boxes through its Hap4L like domain (Hortschansky et al., 2007, 2015; Chen et al., 2011), while Yap5 binds YRE-O using exclusively its bZIP region (this work, Li et al., 2008; Pimentel et al., 2012). Still, the role of CgYap5 in iron starvation is limited to a modest repression of *GRX4* expression, while HapX strongly represses the expression of tens of iron consuming genes in these conditions (Supplementary File S9).

This new role of Yap5 opens the question of the molecular mechanisms which would allow CgYap5 to be a transcriptional repressor when iron is limiting. Several hypotheses can be mentioned, based on the literature. The transcriptional repression by Hap43 requires its Hap4L domain and involves the CCAAT binding complex (Singh et al., 2011). Yap5 only has a vestigial Hap4L domain (Merhej et al., 2015), which was supposed to be non-functional although its activity has actually never been tested so far. Second, Yap7, the ohnolog of Yap5, has been shown to repress transcription by recruiting the general repressor Tup1 (Merhej et al., 2015). Moreover, functional connections between Hap43 and Tup1 have been reported in *C. albicans* (Hsu et al., 2011). Additionally, the shift of Yap5 from an activator to a repressor could be controlled by iron availability, since the binding of iron-sulfur clusters to ScYap5 was shown to significantly change its conformation (Rietzschel et al., 2015).

### Interconnections between CgYap1, CgYap5, CgYap7, and the *C. glabrata* stress response network

Our data led us to propose new roles for Yap1 and Yap7 in the regulation of genes involved, respectively, in heme biosynthesis and in the biogenesis of iron-sulfur cluster proteins. Previously, Yap7 was shown to be a repressor of the heme-containing nitric oxide oxidase Yhb1 in *C. glabrata* and *S. cerevisiae* (Merhej et al., 2015). Our ChIP-seq data indicated that CgYap7 binds many genes encoding enzymes of the cytoplasmic and mitochondrial iron sulfur cluster biogenesis pathway. This result is particularly interesting considering the important role played by the mitochondrial iron sulfur clusters in the sensing of iron availability and in regulating the activity of Yap5, the onholog of Yap7 (Lill et al., 2014; Rietzschel et al., 2015).

**Figure 7:**
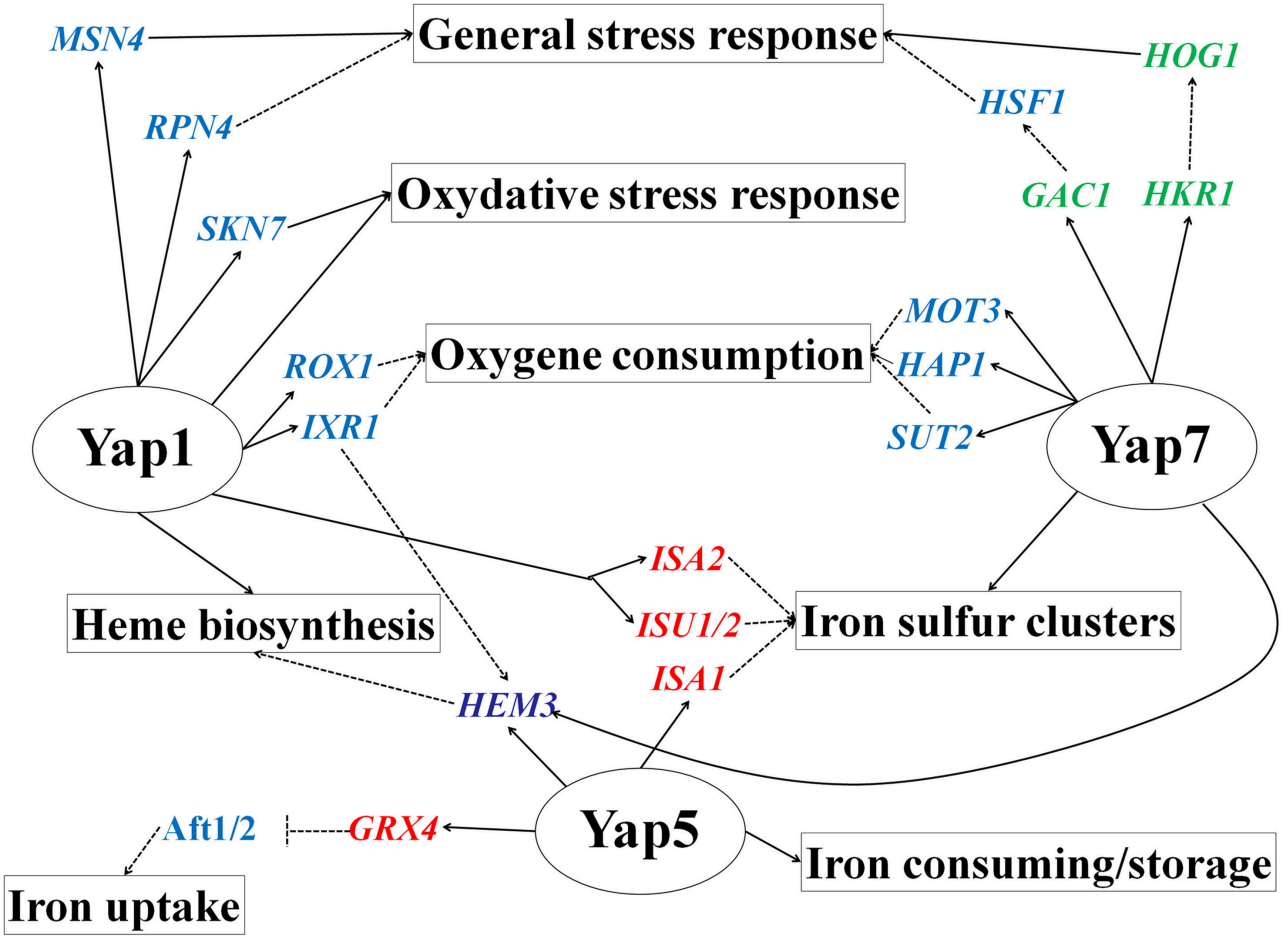
**CgYap1, CgYap5, and CgYap7 define a transcriptional network at the cross-roads between iron homeostasis, oxygen consumption, and stress response**. The plain arrows are regulations which were demonstrated to occur in *C. glabrata*. The dashed arrows are regulations which were demonstrated in *S. cerevisiae* and were just hypothesized by functional annotation transfer in *C. glabrata*. Targeted transcription factors are in blue, kinases and phosphatases in green, iron sulfur cluster binding proteins in red, and heme biosynthetic genes in pink.

In *S. cerevisiae*, there is little evidence of a connection between Yap1 and heme biosynthesis, besides the binding of ScYap1 to *HEM1* (Salin et al., 2008) and the regulation by ScYap1 of the heme-dependent repressors *IXR1* and *ROX1* (Castro-Prego et al., 2010; Caetano et al., 2015). We show here that CgYap1 directly targets four of the eight enzymes involved in this pathway (*HEM1, HEM2, HEM3, HEM15*) and *CgYAP1* deletion clearly impacted the expression of three of them (*HEM1, HEM3*, and *HEM15*). Hence, we defined here a CgYap1/CgYap5/CgYap7 network deeply involved in oxygen and iron sensing by tuning redox homeostasis, heme biosynthesis, iron storage, and iron sulfur cluster metabolism (Figure 7). Each factor has its own specificity (CgYap1 in redox balance and heme biosynthesis, CgYap5 in iron storage and CgYap7 in iron-sulfur clusters biogenesis) but there are some interconnections between them. For instance, CgYap5 and CgYap1 positively control the mitochondrial iron-sulfur cluster proteins maturation factors Isa1 and Isu1/2/Isa2, respectively. Also, the *HEM3* gene, encoding the prophobilinogen deaminase (third step of heme biosynthesis) came out as a “hub” in this network, being positively regulated by CgYap1 and CgYap5 in response to oxidative stress or iron excess, respectively, and repressed by CgYap7 in standard growth conditions (Figure 3). Similarly, *CCP1*, encoding the cytochrome c peroxydase which acts as a heme-based sensor of the mitochondrial oxidative stress (Martins et al., 2013), is positively regulated by CgYap1 and repressed by CgYap7 (Figure 3).

Another interesting feature of CgYap1 and CgYap7 is the strong enrichment for transcription factors among their targets. For instance, they potentially control the expression of several transcription factors known in *S. cerevisiae* for being involved in the regulation of hypoxic genes and oxygen consumption (Rox1 and Ixr1 for CgYap1 and Mot3, Hap1 and Sut2 for CgYap7; Figure 7; Castro-Prego et al., 2010; Gonzalez Siso et al., 2012). They also target some regulators involved in the general Environmental Stress response. This is for instance the case of the Rpn4 and Msn4 transcription factors for CgYap1 or of the Hog1, Hkr1, and Gac1 stress signaling proteins for CgYap7. Additionally, CgYap1 seems to positively control the expression of *SKN7*, which encodes a regulator of oxidative stress response known to cooperate with Yap1 both in *S. cerevisiae* and in *C. glabrata* (Morgan et al., 1997; Lee et al., 1999; Saijo et al., 2010; Roetzer et al., 2011). This suggests that the network described here is tightly connected to other transcriptional responses and that CgYap1 and CgYap7 are master regulators in the *C. glabrata* hierarchy of transcription factors (Jothi et al., 2009; Bhardwaj et al., 2010).

## Author contributions

JM constructed the strains and performed the ChIP-seq and transcriptome analyses. AT performed transcriptome analyses. CB, JP, MA, and SL performed the high-throughput sequencing. GL and JC performed the bioinformatics analyses and the network building. FD designed the experiments and wrote the manuscript.

## Funding

This work was supported by the Agence Nationale pour la Recherche [STRUDYEV, CANDIHUB] and the France Genomique infrastructure as part of the “Investissements d'Avenir” program [ANR-10-INBS-0009].

### Conflict of interest statement

The authors declare that the research was conducted in the absence of any commercial or financial relationships that could be construed as a potential conflict of interest.
