# Supplementary material for: A Network of Paralogous Stress Response Transcription Factors in the Human Pathogen Candida glabrata
Source: Front Microbiol. 2016 May 9;7:645. doi: 10.3389/fmicb.2016.00645 (PMC4860858; doi:10.3389/fmicb.2016.00645)
Supplement: Supplementary file 3 [file Presentation2.PDF]

S. cerevisiae	*C. glabrata*	*C. albicans*
YAP1 (oxidative stress)<br>YAP2 (cadmium)	CAGL0H04631g (YAP1)<br>CAGL0F03069g (YAP2)	CAP1 (oxidative stress)
YAP3	CAGL0K02585g (YAP3a)<br>CAGL0M10087g (YAP3b)	FCR3 (CAP3)
YAP4 (osmotic stress)<br>YAP6 (osmotic stress)	CAGL0M08800g (YAP4/6)	CAP4
YAP5 (iron excess)<br>YAP7	CAGL0K08756g (YAP5)<br>CAGL0F01265g (YAP7)	HAP43 (CAP2) (iron starvation)
YAP8 (arsenic)	Not found	Not found

Supplementary file S1: The YAP family of transcription factors in *S. cerevisiae*, *C. glabrata* and *C. albicans*.

NAME	SEQUENCES (5'=>3')
**Primers for CgYAP1 tagging, deletion, screening and Q-PCR**	
251-CgAP1pro-F1	AAATACCAGAAGAGCCTACAACTTGAAGGTTTCTTAGACACCCAAGGACAAGGACAGAGACAAGAACTAGGAAGTAAAAGC*GGATCC*CCGGGTTAATTAA
252-CgAP1end-F1	TGGCAAAAGCTAAATGTTCGGAAAGGGGTGTCGTGATCAACGCCGATGATGTACAGGTTGCTTTAAATAAGCACATGTCTC*GGATCC*CCGGGTTAATTAA
253-CgAP1ter-R1	TTTTTACATGATTTTAAGTATGCCAGTAACGAGCGTCTTGAATGTGAATTTTGATCTACCGAGATAATATAGACAGGTGAGAATTCGAGCTCGTTTAAAC
254-CgAP1Prom	GGATGATGGGCAGATTTCAC
255-CgAP1ter-Rev	CGATCGCGATAGGTTTTAGT
256-PqCgAP1-F	TGACAACGAAGATGATGACGA
257-PqCgAP1-R	GCCGTTATTCTGTCCCAGAT
**Primers for CgYAP2 tagging, deletion, screening and Q-PCR**	
268-CgAP2pro-F1	GGCTACCACTGATAATTGTGCTAGGTACTCTGAATTGTGGGATAAGCGTGTCAGTGGTACTGGAGAGGACTGGGAAAAGGC*GGATCC*CCGGGTTAATTAA
269-CgAP2end-F1	GCACGGACATAGTACCAAAGCGGCACGTCGTGATCAAAGCGGGAGACCTGCAACGCACCCTCGCAAAGCATATCTCTTCAC*GGATCC*CCGGGTTAATTAA
270-CgAP2ter-R1	TAGTGAATGTGAATTGATTTATGTATGTACTATATACGCCGCTATGCATTATTGAAAAGGGAGAGCTTCCCGTGCAGAGCGAATTCGAGCTCGTTTAAAC
271-CgAP2Prom	GATGTGTCGTCCTGCCAAT
272-CgAP2ter-Rev	GCTTCGATGGTTCCTCACTT
273-PqCgAP2-F	CACGTCAATTGGAGAACGAG
274-PqCgAP2-R	CGTCAGAAGCTCACACCGTA
**Primers for CgYAP3a tagging, deletion, screening and Q-PCR**	
275-CgAP3pro-F1	ACTAGCAAACCATAGACTTTAGTAATTTGTTGTGTCAAGGTTAATACGTGGGAAACGAAAAATAAGTACACTGAACTCTCC*GGATCC*CCGGGTTAATTAA
276-CgAP3end-F1	GCGAAGTTTCTCACAAAAATGGGCCTGCCTACTACAAAAACGTAATAGATACCCTTGTTGAAGAAGCATGCCTAGAAAATC*GGATCC*CCGGGTTAATTAA
277-CgAP3ter-R1	ATCTCCGTAATTAAAAATGGTCCTAAAAAATCGATGTAGATGCCAAGAAACAGACGACTCAAATAGCCACTGTTGTTTTAGAATTCGAGCTCGTTTAAAC
278-CgAP3Prom	GTGACAATCTCAGAGATAACG
279-CgAP3ter-Rev	GGTGACACCGAAGTTACAAT
280-PqCgAP3-F	GCACAGGTTGTGCTTGGTAA
281-PqCgAP3-R	GCTCCAAGTTTTAGGAACAGTCA
**Primers for CgYAP3b tagging, deletion, screening and Q-PCR**	
282-CgAP4pro-F1	TTATTTTCTTTAGTATCTAATAATATATCAGTTTTAAACGTTTTCGGAAATAGACAACAAATAGACAAGTCACACCAAATC*GGATCC*CCGGGTTAATTAA
283-CgAP4end-F1	AGCAGTGTCACCCATCAGGACCAGCTTATTCAAAGCAGAGGATCGATAATTTAGTAAAACAATCAATCGAAAATAATAGGC*GGATCC*CCGGGTTAATTAA
284-CgAP4ter-R1	TTCATAATGTAGAAATCAGTTGCAGAGATTCATTACCTATCGATAATGACAGTTGGTTTCATTATAAGGCAATAATATGGGAATTCGAGCTCGTTTAAAC
285-CgAP4Prom	GCGCTATTGTACAACTAAGGA
286-CgAP4ter-Rev	GGTACAGACGCTCCGAAACA
287-PqCgAP4-F	CCTGATGATGTCCTCTTGACTG

288-PqCgAP4-R	GGTGACACTGCTCCAAACCT

Primers for CgYAP4/6 tagging, deletion, screening and Q-PCR

289-CgAP5pro-F1	TAAGAGATAATGAAACTTCATCAAGTAACAACAGTTCAAATAGTGCGAGTAACGTGATTCACTATATTTCCGGAGATATAC*GGATCC*CCGGGTTAATTAA
290-CgAP5end-F1	TTAACGCTTCAATCAGTGCCAGCAGGCGATTACAAGAGCAACACGAAGAGATTTCAAAATCAATTGCTGGCGAGAAGTCCC*GGATCC*CCGGGTTAATTAA
291-CgAP5ter-R1	AAAGTAAAAAAAAAAAATAAAAAAAAACCTACAATAAATTGTACCTCGATTTGTCTTTTTAGTGCATTCGGAGTTCAGAGGAATTCGAGCTCGTTTAAAC
292-CgAP5Prom	GCTGAAAGACAAAACACTACC
293-CgAP5ter-Rev	CCGGTAACGTAATAGCATGC
294-PqCgAP5-F	GGGCAATGCAAAATAGGAAA
295-PqCgAP5-R	TTTTCGGAAGTGAGGTCCTG

Primers for CgYAP5 tagging, deletion, screening and Q-PCR

296-CgAP6pro-F1	AATGCCCTCACTTGGGTGTAAAATAGTATATATATGTGTGTAGTGCTTAGTAGCTGTTATGTAGTGTGAGGACGATGTGTC*GGATCC*CCGGGTTAATTAA
297-CgAP6end-F1	CTATTTACATGGAAGAACTACCAATGGAGTTTGAAGTCAACTTAAATGATCTATTCCCCTCCCCGAAAAGACAAAGAACAC*GGATCC*CCGGGTTAATTAA
298-CgAP6ter-R1	TTATCGCTGTAGAATAGCTTAATATATTCATAATATCTTATAAATACAAACTTTTACCTGATAGATTCATTGTTCTTAATGAATTCGAGCTCGTTTAAAC
299-CgAP6Prom	GGCATCGCATTGATTACTGC
300-CgAP6ter-Rev	CCACACTAGTAATGTGGAGAT
301-PqCgAP6-F	GGGGAATTGTGGGTTCTGTA
302-PqCgAP6-R	GTGGAGGCTTCGCACTTAAA

Primers for CgYAP7 tagging, deletion, screening and Q-PCR

303-CgAP7pro-F1	TCGGATTTTAGCCTAAACGAAGAAGTCATCGAGGCCATCGGCATCTCGAATCCATATATAATAGACTAGACGTGAAAGCTC*GGATCC*CCGGGTTAATTAA
304-CgAP7end-F1	ATATGGAATACAAGAGAAATTTCCCAACTAACTCAGGAAACTCACCCAGCAACCTGGATAATATACTGGCAAAGCCTGTAC*GGATCC*CCGGGTTAATTAA
305-CgAP7ter-R1	CTGCTTCACATTAACGACTTCAGTTTGATTTGTTAGGCGTTTGTTTTTGATTTTCTAGCCTCTTTTTTATTAAACTGTACGAATTCGAGCTCGTTTAAAC
306-CgAP7Prom	GCACGGACCGCATCAAGAAA
307-CgAP7ter-Rev	GCCTGTAAGTAGAATTATTCTC
308-PqCgAP7-F	GACACAGCATCAGCAACCAC
309-PqCgAP7-R	TGCAGATGTGGCACTACCAT

Primers for screening in the HIS5 (myc-tagging) or TRP1 (knock-out) cassettes

327-TRP1-For	TACGGCATTGATATCGTCCA
328-TRP1-Rev	TCCGCTTACATCAACACCAA
329-HIS5-For	GCAAACCAAAAGGGAGAACA
330-HIS5-Rev	GGACAATTCCCCAACCTTTT

Strain name	Genotype	Origin
**ΔHTU**	his5D/trp1D/ura3D	Kitada et al., 1995
**CgΔAP1**	CAGL0H04631g::TRP1/his5D/trp1D/ura3D	This work
**CgΔAP2**	CAGL0F03069g::TRP1/his5D/trp1D/ura3D	This work
**CgΔAP3a**	CAGL0K02585g::TRP1/his5D/trp1D/ura3D	This work
**CgΔAP3b**	CAGL0M10087g::TRP1/his5D/trp1D/ura3D	This work
**CgΔAP4/6**	CAGL0M08800g::TRP1/his5D/trp1D/ura3D	This work
**CgΔAP5**	CAGL0K08756g::TRP1/his5D/trp1D/ura3D	Merhej et al., 2015
**CgΔAP7**	CAGL0F01265g::TRP1/his5D/trp1D/ura3D	Merhej et al., 2015
**CgAP1-myc**	CAGL0H04631g-13Myc-HIS5/his5D/trp1D/ura3D	This work
**CgAP2-myc**	CAGL0F03069g-13Myc-HIS5/his5D/trp1D/ura3D	This work
**CgAP3a-myc**	CAGL0K02585g-13Myc-HIS5his5D/trp1D/ura3D	This work
**CgAP3b-myc**	CAGL0M10087g-13Myc-HIS5/his5D/trp1D/ura3D	This work
**CgAP4/6-myc**	CAGL0M08800g-13Myc-HIS5/his5D/trp1D/ura3D	This work
**CgΔAP5::pGRB2.1-CgAP5-myc**	CAGL0K08756g::TRP1/his5D/trp1D/ura3D pGRB2.1-HIS-CgAP6-myc	Merhej et al., 2015
**CgΔAP7::pGRB2.1-CgAP7-myc**	CAGL0F01265g::TRP1/his5D/trp1D/ura3D pGRB2.1-HIS-CgAP7-myc	Merhej et al., 2015

Supplementary file S2: Strains and primers used in this study.

Supplementary file S3: Final list of ChIP peaks resulting from the peak calling procedures (see methods).

The genomic location, the identification number, the genes having the peak upstream of their ATG are indicated.

The ChIP peaks with no potential gene targets are located in intergenic regions corresponding to convergent genes.

Chromosome	Start	End	peak ID	Neighbour gene (C strand)	Neighbour gene (W strand)
ChrA	21151	21301	Peak_CgAp7_1	_	CAGL0A00209g
ChrA	30700	31100	Peak_CgAp7_2	CAGL0A00297g	CAGL0A00319g
ChrA	73801	73951	Peak_CgAp1_1	_	CAGL0A00737g
ChrA	186801	187251	Peak_CgAp4-6_1	CAGL0A01826g	_
ChrA	367951	369001	Peak_CgAp4-6_2	CAGL0A03608g	_
ChrA	368451	368751	Peak_CgAp7_3	CAGL0A03608g	_
ChrB	80851	81101	Peak_CgAp1_2	CAGL0B00902g	_
ChrB	129801	130051	Peak_CgAp1_3	CAGL0B01419g	_
ChrB	161401	161851	Peak_CgAp7_4	CAGL0B01727g	CAGL0B01771g
ChrB	204051	204251	Peak_CgAp7_5	_	_
ChrB	224100	224400	Peak_CgAp7_6	CAGL0B02343g	_
ChrB	224201	224351	Peak_CgAp1_4	CAGL0B02343g	_
ChrB	250451	250751	Peak_CgAp1_5	CAGL0B02607g	CAGL0B02629g
ChrB	300151	300901	Peak_CgAp1_6	_	CAGL0B03069g
ChrB	300301	300701	Peak_CgAp7_7	_	CAGL0B03069g
ChrB	341300	341650	Peak_CgAp7_8	CAGL0B03421g	_
ChrB	369401	369651	Peak_CgAp7_9	_	CAGL0B03685g
ChrB	472201	472551	Peak_CgAp7_10	_	CAGL0B04895g
ChrC	71951	72251	Peak_CgAp7_11	_	CAGL0C00693g
ChrC	72000	72250	Peak_CgAP5_1	_	CAGL0C00693g
ChrC	140901	141151	Peak_CgAp7_12	_	CAGL0C01331g
ChrC	182951	183401	Peak_CgAp1_7	CAGL0C01705g	CAGL0C01727g
ChrC	249851	250351	Peak_CgAp1_8	_	CAGL0C02519g

ChrC	249900	250150	Peak_CgAp7_13	_	CAGL0C02519g
ChrC	250001	250251	Peak_CgAp4-6_3	_	CAGL0C02519g
ChrC	281801	282001	Peak_CgAp7_14	_	CAGL0C02827g
ChrC	289551	289801	Peak_CgAp7_15	CAGL0C02893g	CAGL0C02937g
ChrC	289651	290201	Peak_CgAp4-6_4	CAGL0C02893g	CAGL0C02937g
ChrC	321300	321550	Peak_CgAP5_2	_	CAGL0C03223g
ChrC	375701	376351	Peak_CgAp4-6_5	CAGL0C03784g	CAGL0C03828g
ChrC	448351	448551	Peak_CgAp7_16	CAGL0C04785g	CAGL0C04807g
ChrC	489301	489951	Peak_CgAp4-6_6	CAGL0C05137g	CAGL0C05159g
ChrD	25600	25900	Peak_CgAp7_17	_	CAGL0D00198g
ChrD	91651	92001	Peak_CgAp4-6_7	CAGL0D00682g	_
ChrD	141201	141551	Peak_CgAp1_9	_	CAGL0D01265g
ChrD	144001	144201	Peak_CgAp7_18	_	CAGL0D01298g
ChrD	164451	164701	Peak_CgAp1_10	CAGL0D01496g	_
ChrD	199901	200301	Peak_CgAp7_19	_	CAGL0D01914g
ChrD	203801	204051	Peak_CgAp1_11	CAGL0D01936g	CAGL0D01958g
ChrD	220901	221151	Peak_CgAp4-6_8	CAGL0D02134g	CAGL0D02150g
ChrD	288651	288951	Peak_CgAp7_20	CAGL0D02728g	_
ChrD	409001	409301	Peak_CgAp1_12	CAGL0D04114g	CAGL0D04136g
ChrD	519551	520001	Peak_CgAp1_13	CAGL0D05434g	CAGL0D05456g
ChrD	520651	520851	Peak_CgAp1_14	CAGL0D05434g	CAGL0D05456g
ChrD	530501	531151	Peak_CgAp4-6_9	CAGL0D05544g	CAGL0D05566g
ChrD	578251	578551	Peak_CgAp1_15	_	CAGL0D06138g
ChrD	607801	608101	Peak_CgAP5_3	CAGL0D06424g	_
ChrE	127001	127351	Peak_CgAp7_21	_	CAGL0E01353g
ChrE	147001	147251	Peak_CgAp7_22	CAGL0E01529g	_
ChrE	187401	187951	Peak_CgAp4-6_10	CAGL0E01859g	CAGL0E01881g
ChrE	187501	187651	Peak_CgAp7_23	CAGL0E01859g	CAGL0E01881g

ChrE	187651	187801	Peak_CgAp7_24	CAGL0E01859g	CAGL0E01881g
ChrE	199201	199401	Peak_CgAp7_25	CAGL0E01991g	_
ChrE	363651	363951	Peak_CgAp7_26	CAGL0E03828g	CAGL0E03850g
ChrE	369101	369351	Peak_CgAp7_27	CAGL0E03894g	CAGL0E03916g
ChrE	391851	392351	Peak_CgAp7_28	CAGL0E04070g	CAGL0E04092g
ChrE	400851	401201	Peak_CgAp7_29	_	CAGL0E04158g
ChrE	417751	418201	Peak_CgAp7_30	CAGL0E04334g	_
ChrE	436901	437201	Peak_CgAp7_31	CAGL0E04548g	CAGL0E04554g
ChrE	474601	474851	Peak_CgAp7_32	CAGL0E04884g	_
ChrE	566800	567200	Peak_CgAP5_4	CAGL0E05676g	_
ChrE	609351	609801	Peak_CgAp4-6_11	CAGL0E06116g	_
ChrE	609401	609701	Peak_CgAp7_33	CAGL0E06116g	_
ChrE	631751	632151	Peak_CgAp7_34	CAGL0E06270g	CAGL0E06292g
ChrE	663901	664101	Peak_CgAp7_35	_	CAGL0E06644g
ChrE	684201	684851	Peak_CgAp7_36	CAGL0E06688g	_
ChrE	685301	685601	Peak_CgAp7_37	CAGL0E06688g	_
ChrF	70701	71201	Peak_CgAp7_38	CAGL0F00627g	CAGL0F00649g
ChrF	130401	130751	Peak_CgAp7_39	_	CAGL0F01265g
ChrF	178751	179151	Peak_CgAp1_16	CAGL0F01793g	_
ChrF	289001	289151	Peak_CgAp7_40	_	CAGL0F03003g
ChrF	294801	295001	Peak_CgAp7_41	_	CAGL0F03025g
ChrF	401351	401951	Peak_CgAp7_42	CAGL0F04015g	CAGL0F04081g
ChrF	424151	424301	Peak_CgAp1_17	CAGL0F04257g	CAGL0F04279g
ChrF	454151	454601	Peak_CgAp7_43	CAGL0F04521g	CAGL0F04543g
ChrF	454701	455001	Peak_CgAp7_44	CAGL0F04521g	CAGL0F04543g
ChrF	455301	455751	Peak_CgAp7_45	CAGL0F04521g	CAGL0F04543g
ChrF	455551	455851	Peak_CgAp1_18	CAGL0F04521g	CAGL0F04543g
ChrF	481701	481851	Peak_CgAp7_46	CAGL0F04763g	CAGL0F04785g

ChrF	501951	502251	Peak_CgAp7_47	CAGL0F04917g	CAGL0F04939g
ChrF	609701	610251	Peak_CgAp7_48	CAGL0F06061g	CAGL0F06127g
ChrF	609801	610201	Peak_CgAp4-6_12	CAGL0F06061g	CAGL0F06127g
ChrF	698001	698301	Peak_CgAp7_49	CAGL0F07117g	_
ChrF	719051	719401	Peak_CgAp1_19	CAGL0F07359g	_
ChrF	731001	731351	Peak_CgAp1_20	CAGL0F07513g	_
ChrF	789551	789901	Peak_CgAp7_50	CAGL0F07997g	_
ChrF	829201	829351	Peak_CgAp1_21	CAGL0F08305g	_
ChrF	834801	835151	Peak_CgAp2_1	CAGL0F08327g	CAGL0F08371g
ChrF	897901	898201	Peak_CgAp1_22	_	CAGL0F09097g
ChrG	32201	32401	Peak_CgAp1_23	CAGL0G00286g	CAGL0G00308g
ChrG	131951	132201	Peak_CgAp7_51	_	_
ChrG	166951	167401	Peak_CgAp4-6_13	CAGL0G01804g	CAGL0G01826g
ChrG	175351	175501	Peak_CgAp7_52	CAGL0G01947g	_
ChrG	271051	271201	Peak_CgAp7_53	CAGL0G02937g	CAGL0G02959g
ChrG	331601	331801	Peak_CgAp1_24	CAGL0G03421g	_
ChrG	339451	339851	Peak_CgAp7_54	CAGL0G03487g	_
ChrG	371900	372300	Peak_CgAP5_7	CAGL0G03905g	_
ChrG	469151	469801	Peak_CgAp4-6_14	CAGL0G04873g	CAGL0G04895g
ChrG	469251	469451	Peak_CgAp7_55	CAGL0G04873g	CAGL0G04895g
ChrG	495701	495851	Peak_CgAp7_56	CAGL0G05269g	_
ChrG	588751	589001	Peak_CgAp1_25	_	CAGL0G06182g
ChrG	726701	727151	Peak_CgAp4-6_15	CAGL0G07645g	CAGL0G07667g
ChrG	726751	727051	Peak_CgAp7_57	CAGL0G07645g	CAGL0G07667g
ChrG	758251	758801	Peak_CgAp4-6_16	CAGL0G08019g	_
ChrG	760901	761301	Peak_CgAP5_6	CAGL0G08041g	CAGL0G08063g
ChrG	775701	776301	Peak_CgAP5_5	CAGL0G08151g	CAGL0G08173g
ChrG	849501	849801	Peak_CgAp7_58	CAGL0G08844g	CAGL0G08866g

ChrG	950551	950851	Peak_CgAp1_26	_	CAGL0G09977g
ChrH	49201	49501	Peak_CgAp7_59	CAGL0H00484g	_
ChrH	69901	70501	Peak_CgAp1_27	CAGL0H00704g	_
ChrH	69901	70451	Peak_CgAp4-6_17	CAGL0H00704g	_
ChrH	80151	80951	Peak_CgAp4-6_18	CAGL0H00803g	CAGL0H00825g
ChrH	80401	80801	Peak_CgAp7_60	CAGL0H00803g	CAGL0H00825g
ChrH	106801	107051	Peak_CgAp3b_1	CAGL0H01144g	CAGL0H01177g
ChrH	135201	135451	Peak_CgAp1_28	_	CAGL0H01375g
ChrH	247901	248451	Peak_CgAp7_61	_	CAGL0H02739g
ChrH	248101	248401	Peak_CgAp1_29	_	CAGL0H02739g
ChrH	350301	350801	Peak_CgAp7_62	CAGL0H03751g	CAGL0H03773g
ChrH	428501	428801	Peak_CgAp4-6_19	CAGL0H04499g	CAGL0H04521g
ChrH	444551	445001	Peak_CgAp1_30	CAGL0H04631g	CAGL0H04653g
ChrH	554901	555101	Peak_CgAp1_31	CAGL0H05599g	CAGL0H05621g
ChrH	565250	565550	Peak_CgAp7_63	CAGL0H05687g	_
ChrH	586101	586451	Peak_CgAp1_32	CAGL0H05951g	_
ChrH	590651	591251	Peak_CgAp1_33	CAGL0H06017g	CAGL0H06105g
ChrH	590701	591151	Peak_CgAp7_64	CAGL0H06017g	CAGL0H06105g
ChrH	595151	595451	Peak_CgAp7_65	CAGL0H06039g	CAGL0H06105g
ChrH	623601	623901	Peak_CgAp7_66	CAGL0H06303g	_
ChrH	681751	682401	Peak_CgAp4-6_20	CAGL0H06853g	_
ChrH	681751	682501	Peak_CgAp7_67	CAGL0H06853g	_
ChrH	716851	717251	Peak_CgAp1_34	_	CAGL0H07337g
ChrH	716951	717151	Peak_CgAp7_68	_	CAGL0H07337g
ChrH	717451	717851	Peak_CgAp7_69	_	CAGL0H07337g
ChrH	800400	800750	Peak_CgAp7_70	CAGL0H08129g	_
ChrH	804051	806701	Peak_CgAp4-6_21	_	_
ChrH	895351	895501	Peak_CgAp7_71	CAGL0H09130g	_

ChrH	954751	955051	Peak_CgAp4-6_22	CAGL0H09768g	_
ChrH	977551	977751	Peak_CgAp1_35	CAGL0H10010g	_
ChrH	989051	989351	Peak_CgAp1_36	_	CAGL0H10142g
ChrH	1043151	1043601	Peak_CgAp7_72	_	CAGL0H10626g
ChrI	28451	28701	Peak_CgAp1_37	CAGL0I00374g	_
ChrI	37301	37901	Peak_CgAp4-6_23	CAGL0I00484g	_
ChrI	53001	53501	Peak_CgAp1_38	CAGL0I00726g	CAGL0I00748g
ChrI	93401	93751	Peak_CgAp1_39	CAGL0I01100g	CAGL0I01122g
ChrI	96101	96701	Peak_CgAp1_40	_	CAGL0I01166g
ChrI	109401	109551	Peak_CgAp7_73	CAGL0I01320g	_
ChrI	230951	231851	Peak_CgAp7_74	_	CAGL0I02574g
ChrI	231001	232001	Peak_CgAp4-6_24	_	CAGL0I02574g
ChrI	231101	231451	Peak_CgAp1_41	_	CAGL0I02574g
ChrI	374400	374750	Peak_CgAp7_75	CAGL0I04224g	CAGL0I04246g
ChrI	419901	420451	Peak_CgAp4-6_25	CAGL0I04686g	CAGL0I04708g
ChrI	444901	445451	Peak_CgAp1_42	CAGL0I04862g	CAGL0I04884g
ChrI	530551	530751	Peak_CgAp1_43	CAGL0I05610g	CAGL0I05632g
ChrI	659901	661001	Peak_CgAp4-6_26	CAGL0I06787g	CAGL0I06809g
ChrI	660101	660601	Peak_CgAp7_76	CAGL0I06787g	CAGL0I06809g
ChrI	663751	664001	Peak_CgAp3b_2	_	_
ChrI	715551	715851	Peak_CgAp7_77	CAGL0I07425g	CAGL0I07447g
ChrI	729501	729751	Peak_CgAp7_78	CAGL0I07535g	CAGL0I07557g
ChrI	796151	796751	Peak_CgAp7_79	_	CAGL0I08195g
ChrI	844801	845201	Peak_CgAp1_44	CAGL0I08635g	CAGL0I08701g
ChrI	873251	873651	Peak_CgAp2_2	CAGL0I08987g	_
ChrI	873251	873651	Peak_CgAp7_80	CAGL0I08987g	_
ChrI	881501	881701	Peak_CgAp7_81	CAGL0I09086g	_
ChrI	940551	941201	Peak_CgAp7_82	CAGL0I09834g	CAGL0I09856g

ChrI	966851	967601	Peak_CgAp7_83	CAGL0I10098g	_
ChrI	984751	985051	Peak_CgAp1_45	CAGL0I10147g	CAGL0I10200g
ChrI	984751	985001	Peak_CgAp7_84	CAGL0I10147g	CAGL0I10200g
ChrJ	77551	77901	Peak_CgAp7_85	_	_
ChrJ	210351	210601	Peak_CgAp7_86	CAGL0J02090g	CAGL0J02112g
ChrJ	284351	285001	Peak_CgAp4-6_27	_	CAGL0J02948g
ChrJ	284701	284901	Peak_CgAp7_87	_	CAGL0J02948g
ChrJ	377201	377801	Peak_CgAp4-6_28	CAGL0J03960g	CAGL0J04004g
ChrJ	377251	377801	Peak_CgAp7_88	CAGL0J03960g	CAGL0J04004g
ChrJ	383651	383851	Peak_CgAp1_46	CAGL0J04026g	CAGL0J04048g
ChrJ	387401	387651	Peak_CgAp7_89	CAGL0J04092g	CAGL0J04136g
ChrJ	435251	435701	Peak_CgAp4-6_29	CAGL0J04554g	_
ChrJ	496001	496501	Peak_CgAp7_90	CAGL0J05159g	_
ChrJ	687001	687601	Peak_CgAp7_91	CAGL0J07106g	CAGL0J07128g
ChrJ	738200	738550	Peak_CgAp7_92	CAGL0J07568g	CAGL0J07590g
ChrJ	740151	740501	Peak_CgAp1_47	_	CAGL0J07612g
ChrJ	870651	871101	Peak_CgAp4-6_30	CAGL0J08800g	CAGL0J08822g
ChrJ	870851	871051	Peak_CgAp7_93	CAGL0J08800g	CAGL0J08822g
ChrJ	953151	953551	Peak_CgAp7_94	CAGL0J09680g	_
ChrJ	953201	953851	Peak_CgAp1_48	CAGL0J09680g	_
ChrJ	953251	953751	Peak_CgAP5_8	CAGL0J09680g	_
ChrJ	972301	972801	Peak_CgAp4-6_31	_	CAGL0J09922g
ChrJ	1028551	1028901	Peak_CgAp1_49	_	CAGL0J10494g
ChrK	86200	86600	Peak_CgAp1_50	_	CAGL0K00803g
ChrK	155051	155401	Peak_CgAp1_51	CAGL0K01727g	_
ChrK	268701	269801	Peak_CgAp4-6_32	_	CAGL0K03003g
ChrK	268801	269751	Peak_CgAp7_95	_	CAGL0K03003g
ChrK	269851	270401	Peak_CgAp4-6_33	_	CAGL0K03003g

ChrK	269951	270151	Peak_CgAp7_96	_	CAGL0K03003g
ChrK	329951	330301	Peak_CgAp1_52	CAGL0K03531g	CAGL0K03553g
ChrK	495001	495501	Peak_CgAp4-6_34	CAGL0K05071g	CAGL0K05093g
ChrK	495151	495401	Peak_CgAp1_53	CAGL0K05071g	CAGL0K05093g
ChrK	524601	524801	Peak_CgAp7_97	_	CAGL0K05357g
ChrK	530701	531251	Peak_CgAp4-6_35	_	_
ChrK	583501	583751	Peak_CgAp1_54	CAGL0K05973g	CAGL0K05995g
ChrK	608451	609201	Peak_CgAp1_55	CAGL0K06259g	_
ChrK	609401	609751	Peak_CgAp1_56	CAGL0K06259g	_
ChrK	684351	684751	Peak_CgAp7_98	_	CAGL0K07007g
ChrK	710151	710601	Peak_CgAp4-6_36	CAGL0K07205g	_
ChrK	813351	813801	Peak_CgAp7_99	CAGL0K08162g	CAGL0K08184g
ChrK	813451	813801	Peak_CgAp1_57	CAGL0K08162g	CAGL0K08184g
ChrK	838301	838651	Peak_CgAp4-6_37	CAGL0K08294g	CAGL0K08316g
ChrK	860200	860350	Peak_CgAp7_100	CAGL0K08514g	CAGL0K08536g
ChrK	883601	883751	Peak_CgAp1_58	_	CAGL0K08800g
ChrK	924551	925001	Peak_CgAp1_59	CAGL0K09350g	_
ChrK	924551	924901	Peak_CgAp7_101	CAGL0K09350g	_
ChrK	927851	928101	Peak_CgAp1_60	CAGL0K09372g	_
ChrK	1059201	1059951	Peak_CgAp1_61	CAGL0K10868g	CAGL0K10890g
ChrK	1071251	1071601	Peak_CgAp7_102	CAGL0K10956g	CAGL0K10978g
ChrK	1109450	1109700	Peak_CgAp7_103	CAGL0K11396g	_
ChrK	1145951	1146301	Peak_CgAp1_62	CAGL0K11858g	CAGL0K11880g
ChrK	1146050	1146250	Peak_CgAp3b_3	CAGL0K11858g	CAGL0K11880g
ChrL	103701	103851	Peak_CgAp1_63	_	_
ChrL	131901	132201	Peak_CgAP5_9	CAGL0L01089g	CAGL0L01111g
ChrL	204701	205451	Peak_CgAp7_104	_	CAGL0L01793g
ChrL	239551	239801	Peak_CgAp1_64	CAGL0L02013g	_

ChrL	277551	278201	Peak_CgAp4-6_38	CAGL0L02431g	_
ChrL	277601	278101	Peak_CgAp7_105	CAGL0L02431g	_
ChrL	285651	286051	Peak_CgAp7_106	CAGL0L02453g	_
ChrL	290901	291651	Peak_CgAp4-6_39	CAGL0L02453g	_
ChrL	308400	308600	Peak_CgAp7_107	CAGL0L02607g	_
ChrL	345851	346051	Peak_CgAp1_65	CAGL0L02937g	CAGL0L02959g
ChrL	417301	417551	Peak_CgAp1_66	CAGL0L03608g	CAGL0L03630g
ChrL	537001	537301	Peak_CgAp1_67	CAGL0L04642g	CAGL0L04664g
ChrL	542601	542851	Peak_CgAp7_108	_	CAGL0L04730g
ChrL	588451	589001	Peak_CgAp1_68	CAGL0L05258g	CAGL0L05280g
ChrL	601651	601801	Peak_CgAp1_69	CAGL0L05456g	CAGL0L05478g
ChrL	634251	634601	Peak_CgAp1_70	CAGL0L05742g	_
ChrL	636851	637251	Peak_CgAp7_109	CAGL0L05742g	_
ChrL	641701	642201	Peak_CgAp7_110	CAGL0L05786g	CAGL0L05830g
ChrL	685551	685851	Peak_CgAp1_71	CAGL0L06072g	CAGL0L06094g
ChrL	687701	688451	Peak_CgAp4-6_40	CAGL0L06072g	CAGL0L06094g
ChrL	687701	688401	Peak_CgAp7_111	CAGL0L06072g	CAGL0L06094g
ChrL	688051	688551	Peak_CgAp1_72	CAGL0L06072g	CAGL0L06094g
ChrL	719351	720201	Peak_CgAp2_3	CAGL0L06374g	CAGL0L06402g
ChrL	719751	720251	Peak_CgAp7_112	CAGL0L06374g	CAGL0L06402g
ChrL	752051	752351	Peak_CgAp7_113	CAGL0L06622g	CAGL0L06666g
ChrL	752701	753401	Peak_CgAp7_114	_	CAGL0L06666g
ChrL	753451	754101	Peak_CgAp7_115	_	CAGL0L06666g
ChrL	1061651	1062201	Peak_CgAp7_116	_	CAGL0L09911g
ChrL	1061701	1062201	Peak_CgAp4-6_41	_	CAGL0L09911g
ChrL	1062951	1063101	Peak_CgAp7_117	_	CAGL0L09911g
ChrL	1108551	1108801	Peak_CgAp1_73	CAGL0L10340g	_
ChrL	1169551	1169801	Peak_CgAp7_118	CAGL0L10912g	CAGL0L10934g

ChrL	1235601	1235901	Peak_CgAp1_74	_	CAGL0L11572g
ChrL	1394201	1394501	Peak_CgAp7_119	_	CAGL0L13046g
ChrL	1425001	1425451	Peak_CgAp7_120	CAGL0L13288g	CAGL0L13299g
ChrM	3401	3951	Peak_CgAp4-6_42	_	_
ChrM	195851	196501	Peak_CgAp4-6_43	CAGL0M01716g	CAGL0M01738g
ChrM	195900	196400	Peak_CgAp7_121	CAGL0M01716g	CAGL0M01738g
ChrM	226551	227101	Peak_CgAp7_122	CAGL0M01870g	_
ChrM	241551	241901	Peak_CgAp7_123	CAGL0M01958g	_
ChrM	294251	294801	Peak_CgAp4-6_44	CAGL0M02541g	_
ChrM	302551	303201	Peak_CgAp1_75	CAGL0M02607g	CAGL0M02629g
ChrM	614501	615001	Peak_CgAp1_76	_	CAGL0M05819g
ChrM	738701	739401	Peak_CgAp4-6_45	CAGL0M07293g	_
ChrM	738901	739301	Peak_CgAp7_124	CAGL0M07293g	_
ChrM	749301	749501	Peak_CgAp7_125	_	_
ChrM	791851	792201	Peak_CgAp7_126	CAGL0M07920g	_
ChrM	849101	849251	Peak_CgAp7_127	_	_
ChrM	861300	861500	Peak_CgAp7_128	CAGL0M08624g	CAGL0M08646g
ChrM	873851	874351	Peak_CgAp7_129	_	CAGL0M08800g
ChrM	889651	889851	Peak_CgAp1_77	CAGL0M08910g	CAGL0M08932g
ChrM	922351	922651	Peak_CgAp7_130	CAGL0M09207g	CAGL0M09229g
ChrM	1004001	1004351	Peak_CgAp1_78	_	CAGL0M10087g
ChrM	1161001	1161651	Peak_CgAp4-6_46	CAGL0M11660g	_
ChrM	1164451	1164901	Peak_CgAp1_79	CAGL0M11682g	CAGL0M11704g
ChrM	1167451	1167801	Peak_CgAp7_131	CAGL0M11726g	CAGL0M11748g
ChrM	1181801	1181951	Peak_CgAp1_80	_	CAGL0M11902g
ChrM	1194251	1194601	Peak_CgAp1_81	CAGL0M11990g	_
ChrM	1198551	1199101	Peak_CgAp7_132	CAGL0M12012g	CAGL0M12034g
ChrM	1205651	1206201	Peak_CgAp7_133	_	_

ChrM	1240851	1241051	Peak_CgAp1_82	CAGL0M12430g	CAGL0M12452g
ChrM	1298351	1298601	Peak_CgAp1_83	CAGL0M13189g	CAGL0M13211g
ChrM	1333201	1333501	Peak_CgAp1_84	CAGL0M13519g	CAGL0M13541g

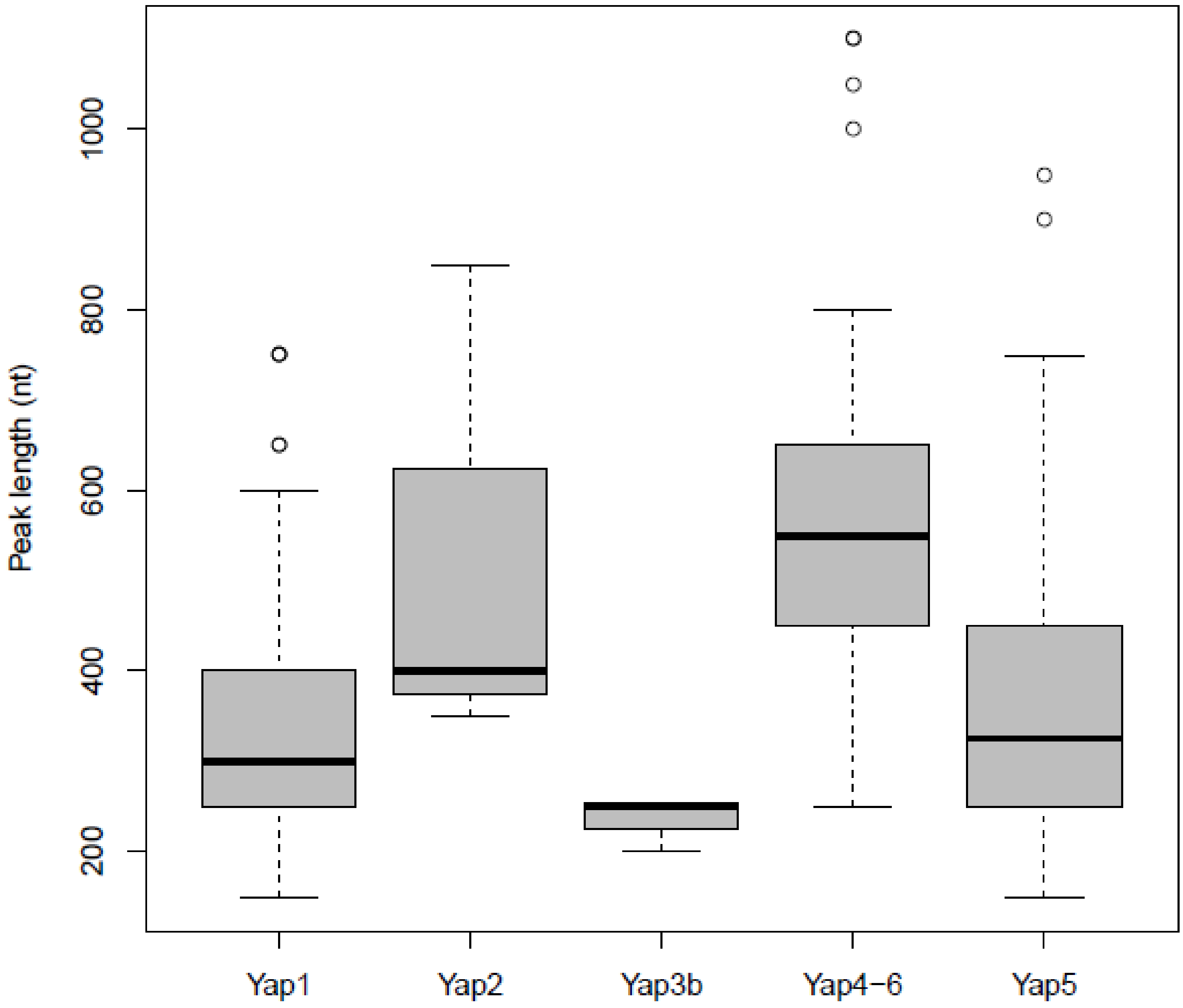

Supplementary file S4: Distribution of the size of the selected ChIP peaks.

Supplementary file S5: Complete results of TFBS predictions using peak motif. For each CgYap, the sequence logo and the type of motif are indicated. The number of occurrences of the motif in the ChIP list is indicated below the corresponding logo. Motifs have been sorted from the most frequent to the least.

YAP1

Motif | Logo/ number of sites | Motif type

Oligos_8nt_mkv2_m1

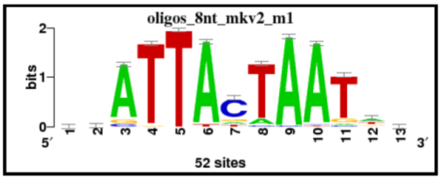

YRE-O

Oligos_7nt_mkv2_m1

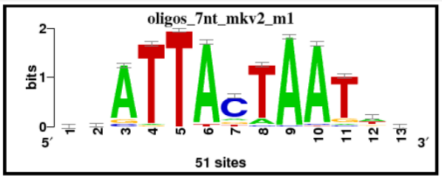

YRE-O

Oligos_6nt_mkv2_m1

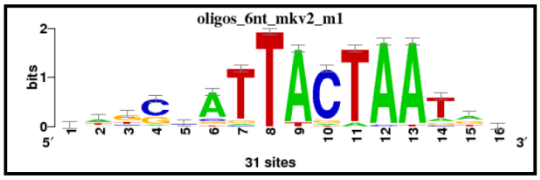

YRE-O

Oligos_8nt_mkv2_m3

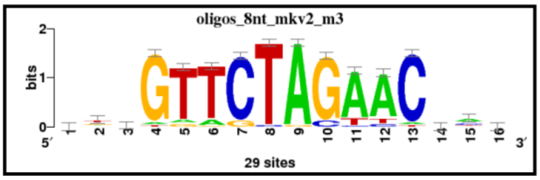

HSE

YAP3b

Motif	Logo/ number of sites	Motif type

Oligos_8nt_mkv1_m1

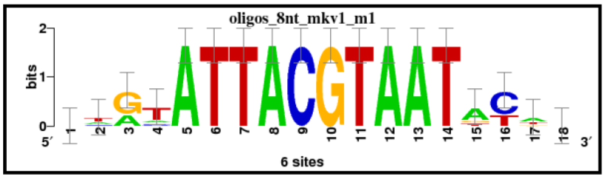

YRE-A

Oligos_7nt_mkv1_m2

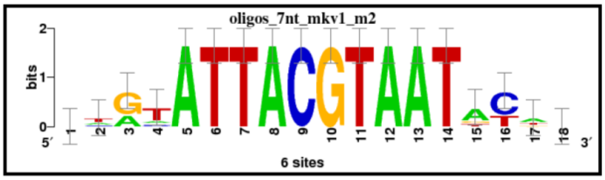

YRE-A

YAP4/6

Motif	Logo/ number of sites	Motif type
Oligos_6nt_mkv2_m1	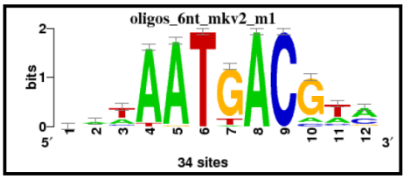	SKO1/CREB
Oligos_7nt_mkv2_m1	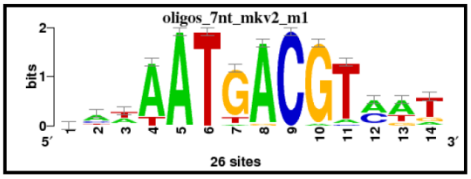	SKO1/CREB
Oligos_8nt_m1	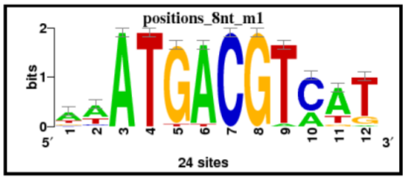	SKO1/CREB
Oligos_8nt_mkv2_m2	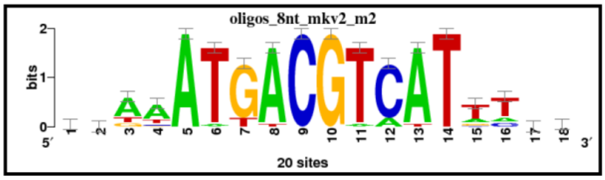	SKO1/CREB
Oligos_7nt_m2	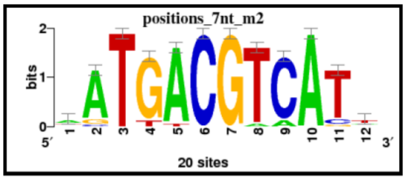	SKO1/CREB
Oligos_7nt_mkv2_m5	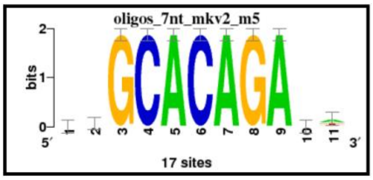	Unknown
Oligos_8nt_mkv2_m5	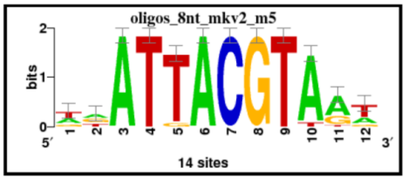	YRE-A
Oligos_7nt_mkv2_m3	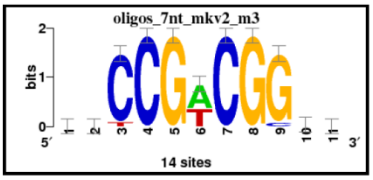	Zinc Finger like

YAP5

Motif	Logo/ number of sites	Motif type
Oligos_7nt_mkv1_m1	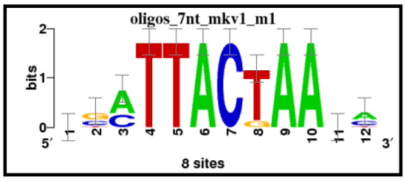	YRE-O
Oligos_8nt_mkv1_m2	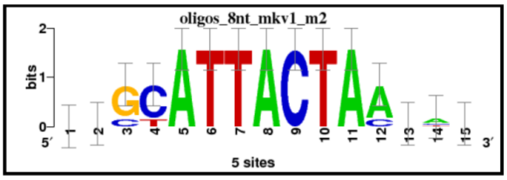	YRE-O
Oligos_8nt_mkv1_m4	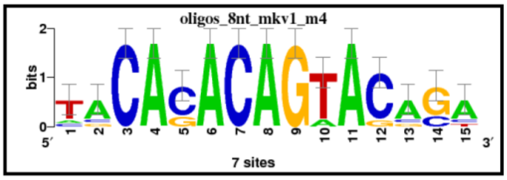	Unknown
Oligos_8nt_mkv1_m3	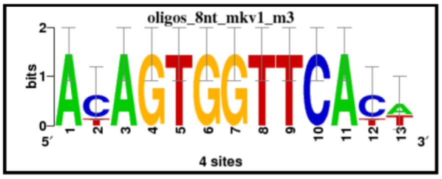	Unknown

YAP7

Motif	Logo/ number of sites	Motif type
Positions_7nt_m1	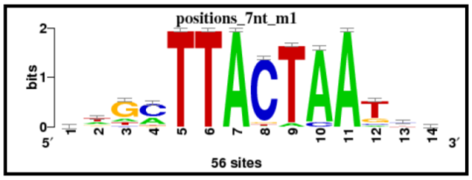	YRE-O
Oligos_6nt_mkv2_m3	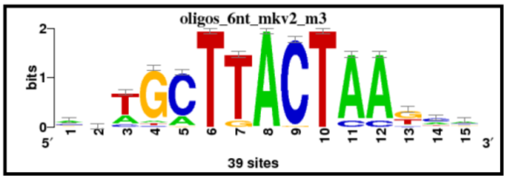	YRE-O
Oligos_6nt_mkv2_m1	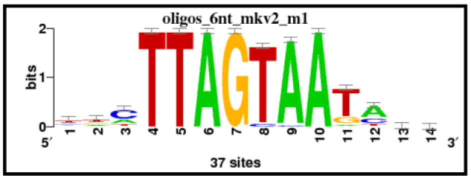	YRE-O
Oligos_7nt_mkv2_m1	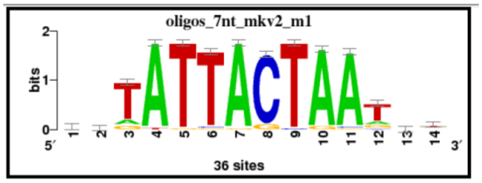	YRE-O
Oligos_8nt_mkv2_m4	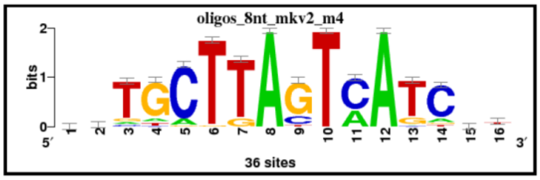	YRE-O
Oligos_8nt_mkv2_m1	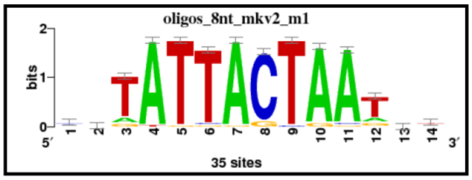	YRE-O
Oligos_7nt_mkv2_m2	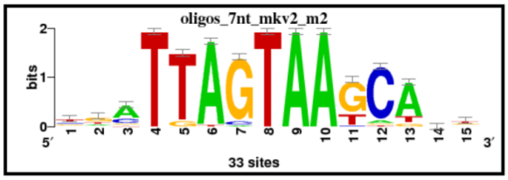	YRE-O
Oligos_7nt_mkv2_m3	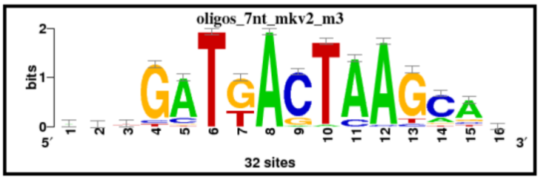	YRE-O

YAP7

Motif	Logo/ number of sites	Motif type
Oligos_7nt_mkv2_m5	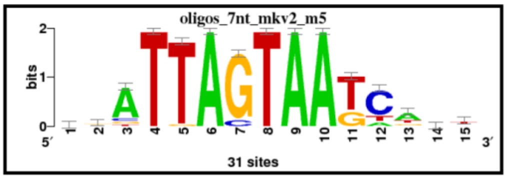	YRE-O
Positions_8nt_m1	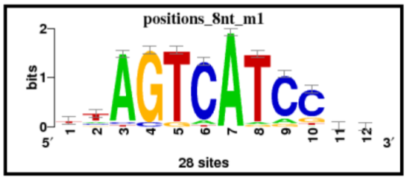	GCN4/BAS1
Positions_6nt	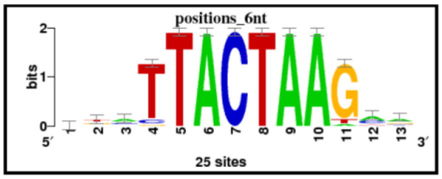	YRE-O

CgYAP1- ChIP

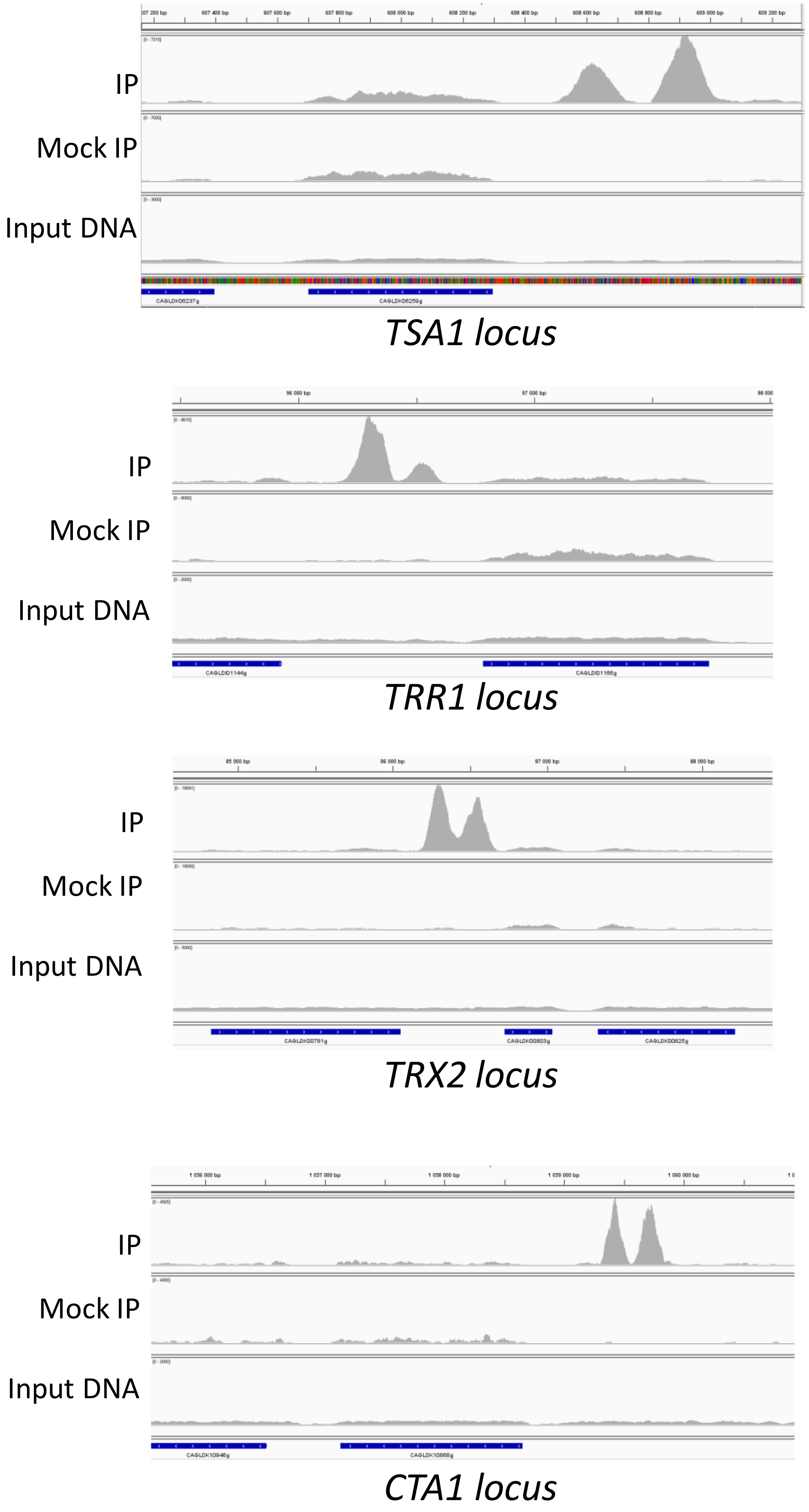

IP
Mock IP
Input DNA
TSA1 locus
IP
Mock IP
Input DNA
TRR1 locus
IP
Mock IP
Input DNA
TRX2 locus
IP
Mock IP
Input DNA
CTA1 locus

CgYAP7-ChIP

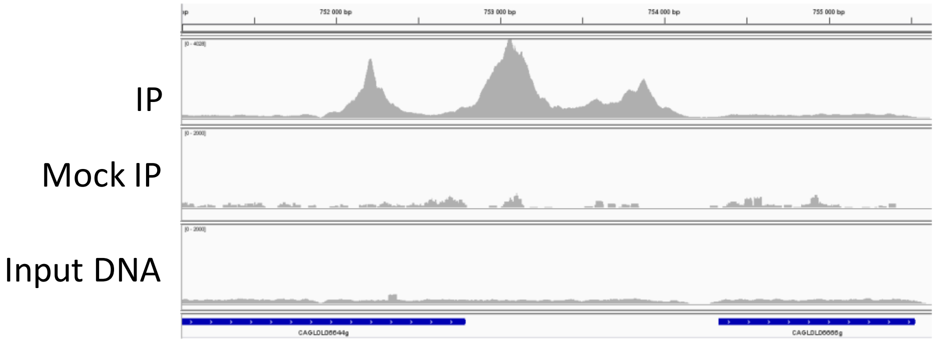

YHB1 locus

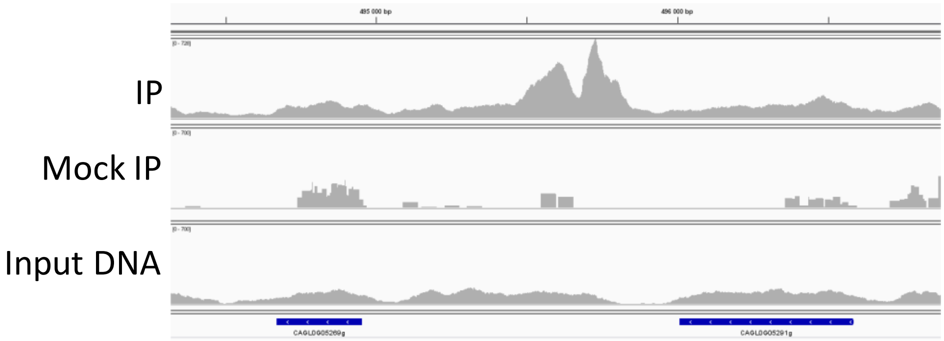

FMP16 locus

Supplementary file S6 : Examples of multiple binding sites. The ChIP-seq results are visualized using Integrative Genomic Viewer. The IGV scales have been normalized so that the IP, Mock and Input lanes have the same baseline.

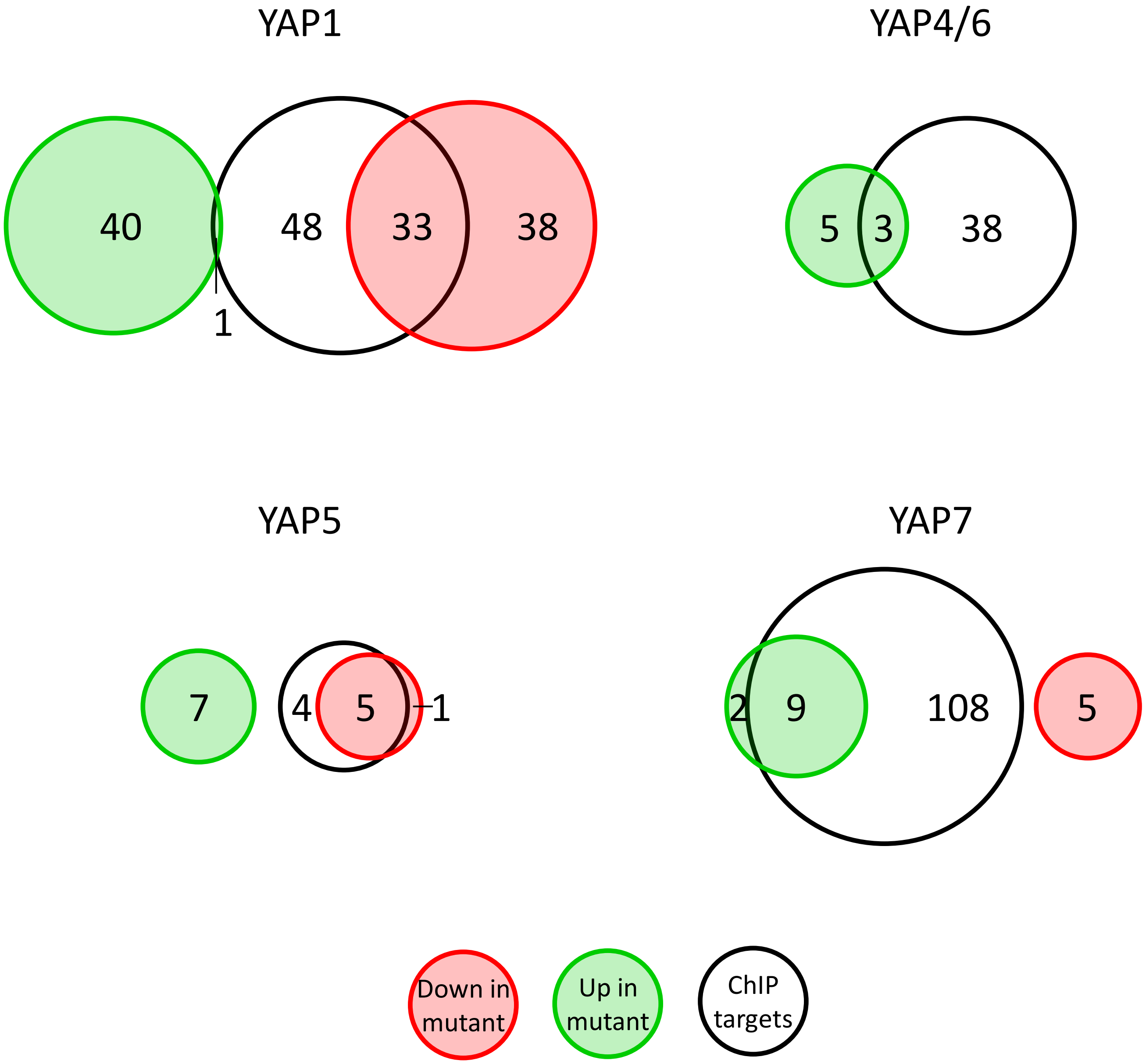

Supplementary file S7 : Venn diagrams of the overlap between the ChIP targets and the transcriptome effects of the *CgYAP* deletions. The black circles are the ChIP targets, the green and red ones represent the genes being respectively up-regulated or down-regulated in the mutant compared with the wild type.

	BPS (This work)		Selenite (in preparation)		Iron stress (This work)	
	pvalue	FDR (%)	*pvalue*	FDR (%)	*pvalue*	FDR (%)
CgYap1	*0,14*	19	***0***	**0**	***0***	**0,55**
CgYap4/6	*0,46*	47	*0,04*	4,85	*0,08*	6,22
CgYap5	***0***	**0**	*0,02*	0,4	***0***	**0,25**
CgYap7	*0,16*	42	*0,01*	3,55	*0,02*	5,88

	Sorbic acid (jandric et al.2013)		Fluconazole (Kuo et al., 2010)		glucose starvation (Roetzer et al., 2008)	
	pvalue	FDR (%)	*pvalue*	FDR (%)	*pvalue*	FDR (%)
CgYap1	***0***	**0,83**	*0,16*	54	*0,1*	24
CgYap4/6	*0,04*	8	*0,98*	97	*0,47*	47
CgYap5	*0,28*	32	*0,38*	54	*0,21*	19
CgYap7	*0,6*	56	*0*	1,1	*0*	14

	H2O2 (Wapinski et al., 2010)		Menadione (Roetzer et al., 2011)		H2O2 (Roetzer et al., 2011)	
	pvalue	FDR (%)	*pvalue*	FDR (%)	*pvalue*	FDR (%)
CgYap1	***0***	**0**	*0,02*	8	***0***	**0**
CgYap4/6	*0,33*	43	*0,65*	99	*0,95*	94
CgYap5	*0,93*	98	*0,3*	70	*0,91*	94
CgYap7	***0***	**0,2**	*0,97*	92	*0,57*	81

	Heat shock (Wapinski et al., 2010)		NaCl (Roetzer et al., 2008)		Na Cl (Wapinski et al., 2010)	
	pvalue	FDR (%)	*pvalue*	FDR (%)	*pvalue*	FDR (%)
CgYap1	***0***	**0,88**	*0,01*	5,35	*0,01*	9
CgYap4/6	*0,83*	83	*0,08*	12	*0,03*	4,7
CgYap5	*0,07*	6,8	*0,3*	33	*0,21*	15
CgYap7	*0,05*	12	*0,94*	84	*0,02*	5,7

	Heat shock (Roetzer et al., 2008)	
	pvalue	FDR (%)
CgYap1	*0,81*	100
CgYap4/6	*0,77*	81
CgYap5	*0,73*	92
CgYap7	*0,19*	79

Supplementary file S8: Complete GSEA results. The publications used are indicating in the corresponding headers. The detailed references can be found in the reference list of the main text. The significant enrichments (FDR<1%) are underlined in red (enrichment in the up-regulated genes) or in green (enrichment in the down-regulated genes).

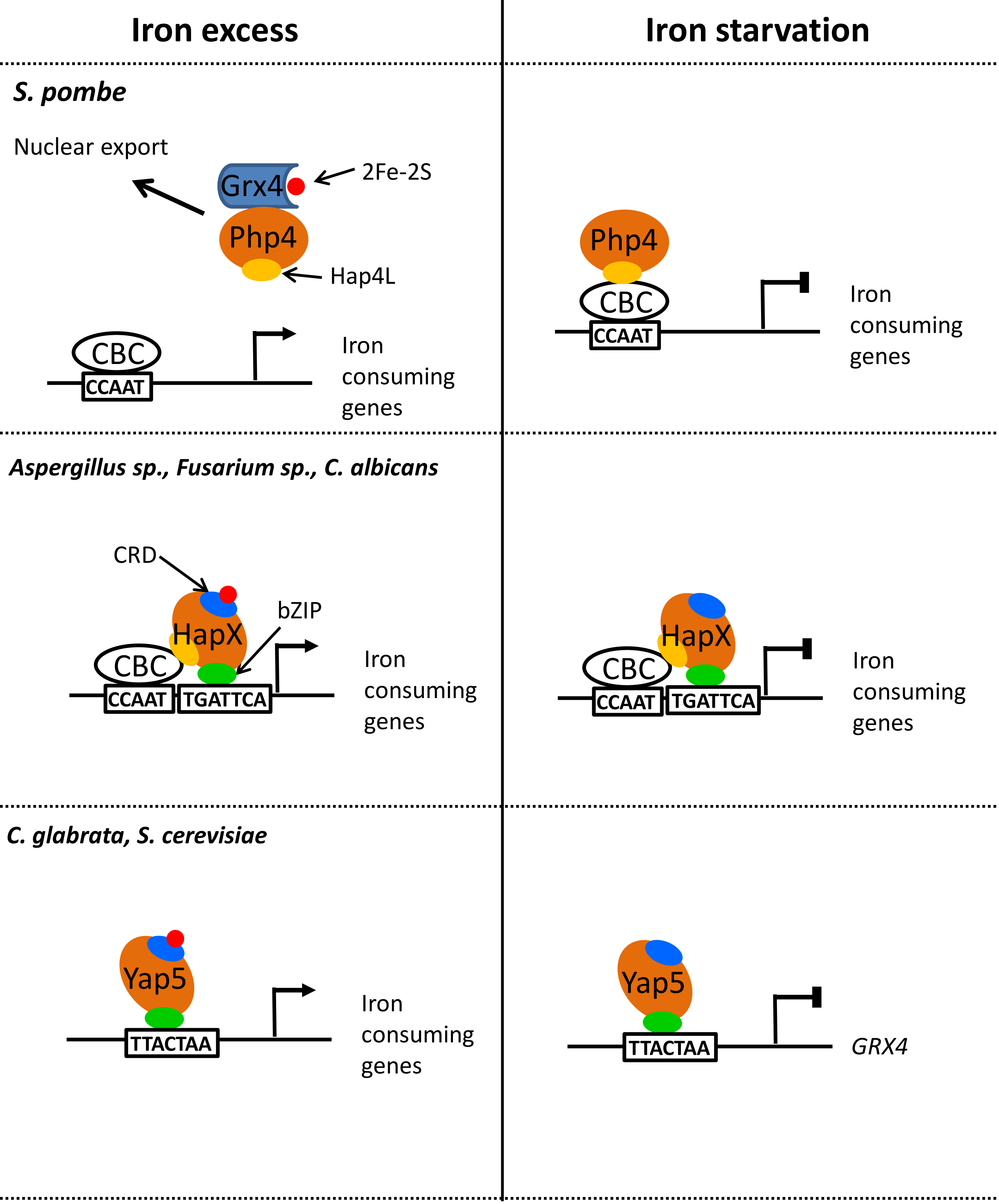

Iron excess
Iron starvation
S. pombe
Nuclear export
Grx4
2Fe-2S
Php4
Hap4L
CBC
CCAAT
Iron consuming genes
Aspergillus sp., Fusarium sp., C. albicans
CRD
HapX
bZIP
TGATTCA
C. glabrata, S. cerevisiae
Yap5
TTACTAA
GRX4

Supplementary file S9 : Evolution of the regulation of iron homeostasis by Hap4L/bZip transcription factors. In *Schizosaccharomyces pombe*, Php4 plays an important role in the iron starvation response by repressing the iron consuming genes through its interaction with the CCAAT Binding Complex (CBC) which is mediated by its Hap4Like domain (Hap4L) (reviewed in (Labbe *et al.*, 2013)). In iron excess conditions, Php4 is exported from the nucleus due to its interaction with the iron-sulfur cluster containing version of the glutaredoxin Grx4 (Labbe *et al.*, 2013). In *S. cerevisiae*, Yap5 is a major regulator of the iron stress response which activates iron consuming genes (Li *et al.*, 2008; Li *et al.*, 2011; Pimentel *et al.*, 2012). Yap5 only has a vestigial Hap4L domain, which role in Yap5 function has not been tested yet (Merhej *et al.*, 2015). Yap5 interacts directly with DNA through its bZIP domain and with iron-sulfur clusters through a conserved Cysteine Rich Domain (CRD) (This work, (Li *et al.*, 2008; Pimentel *et al.*, 2012; Rietzschel *et al.*, 2015)). Yap5 also moderately contributes to the iron starvation response by repressing the expression of *GRX4* (this work). Yap5 is more expressed in iron limiting conditions than in iron excess (this work). Interestingly enough, the situation in filamentous ascomycetes (e.g. *Aspergillus* or *Fusarium* species) and in *C. albicans* is an intermediate between *S. pombe* and *S. cerevisiae*. HapX plays an important dual role in activating the iron stress response and in repressing the same genes in iron starvation (Gsaller *et al.*, 2014; Hortschansky *et al.*, 2007; Hsu *et al.*, 2011; Singh *et al.*, 2011). HapX directly interacts with iron sulfur clusters through a CRD and is transcriptionally repressed in high iron conditions (Gsaller *et al.*, 2014). HapX contains a functional Hap4L-bZIP bipartite domain and cooperate with CBC to bind a bipartite DNA motif (Chen *et al.*, 2011; Gsaller *et al.*, 2014; Hortschansky *et al.*, 2015; Singh *et al.*, 2011)

(A)

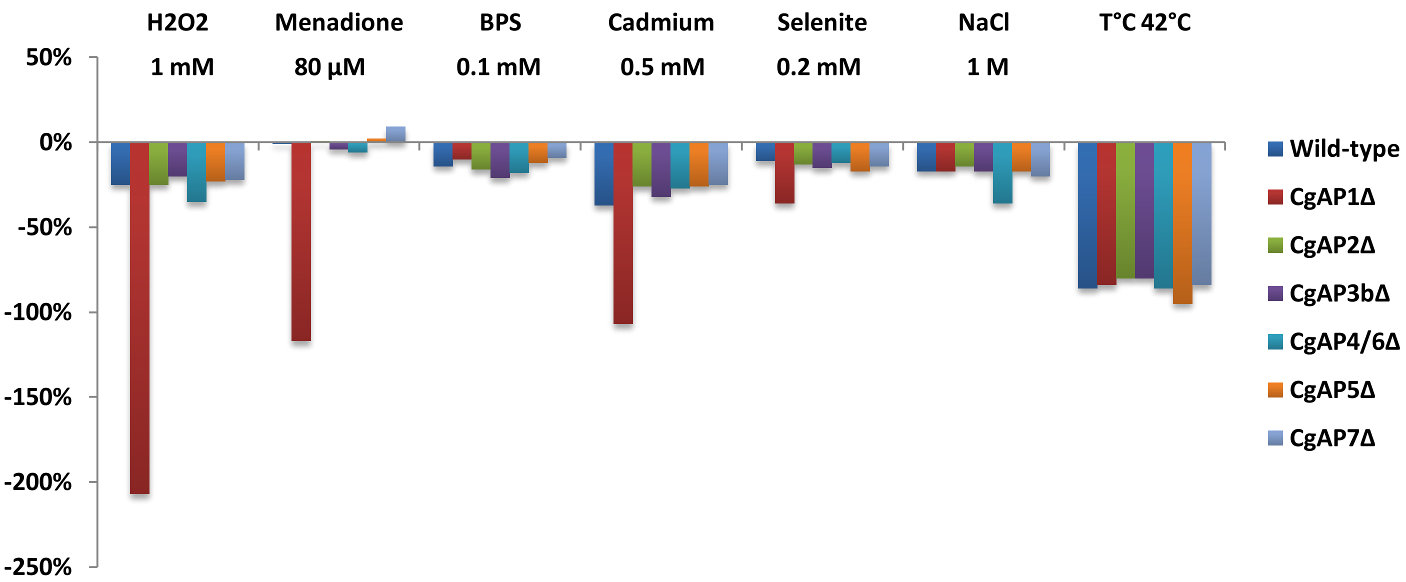

(B)

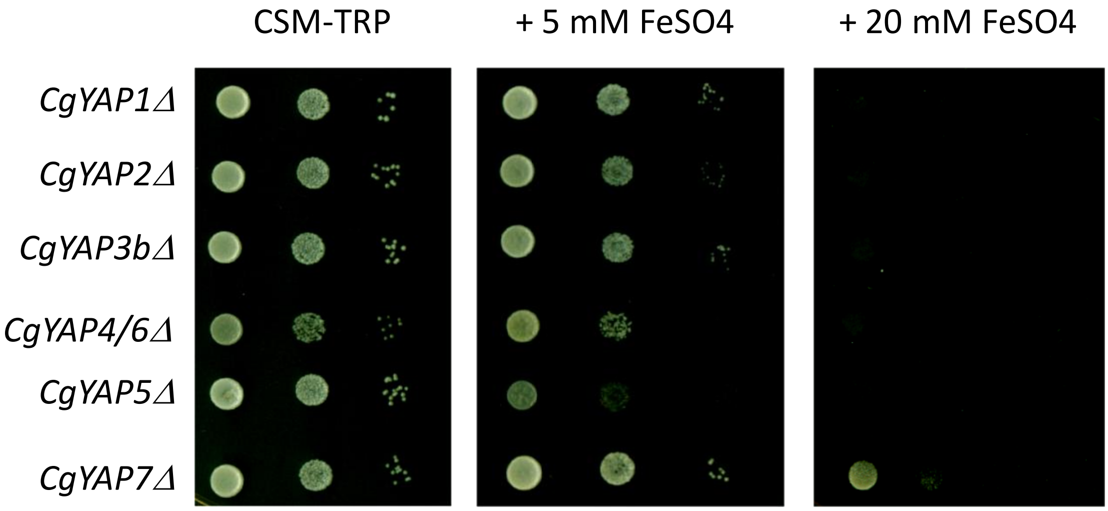

Supplementary file S10: (A) Phenotypic profiling of the *CgYAP* mutant strains in liquid media. Cultures were grown until OD= 0.5 in YPD and then splitted in two. The indicated stresses were applied on one of the two cultures and the OD was measured every thirty minutes for three hours. The indicated values are the percentages of decrease of the growth rates of the stressed cultures, as compared to the growth rates of the untreated cultures (i.e. a value of -100% means that stress exposure divided the growth rate by two). **(B)** Spot assays testing the sensitivity of the *CgYAP* mutants to iron excess. Cells were grown in liquid media until they reached OD=0.8. Droplets of pure cultures and of 1/100 and 1/10000 dilutions were then spotted on CSM-TRP plates supplemented with 0, 5 or 20 mM of iron sulfate. All experiments were replicated at least once.

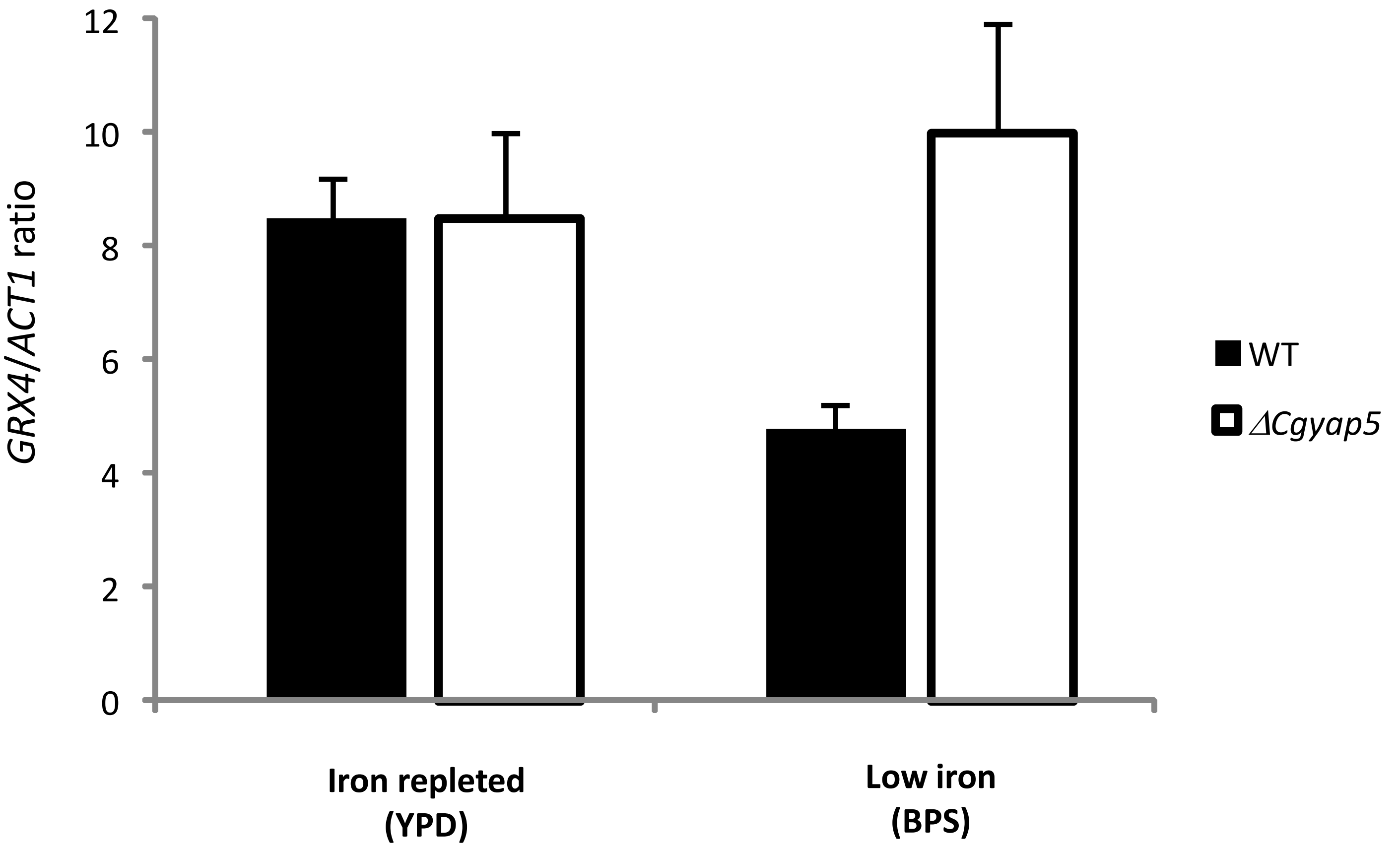

Supplementary file S11: RT-Q-PCR validation of the repressor effect of CgYap5 on *GRX4* under iron starvation conditions, shown in figure 5. RT-Q-PCR experiments were performed exactly as described in (Merhej et al., 2015). The relative expression of *GRX4* was calculated as the difference in the abundance between the transcripts of this gene compared to the transcripts of the *ACT1* gene, used as an endogeneous reference, based on the ΔCt method following the formula '$\mathit{Efficiency}^{(\text{Ct target} - \text{Ct Act1})}$'.
